# Widespread cryptic variation in genetic architecture between the sexes

**DOI:** 10.1002/evl3.245

**Published:** 2021-07-03

**Authors:** Wouter van der Bijl, Judith E. Mank

**Affiliations:** ^1^ Department of Zoology University of British Columbia Vancouver BC V6T 1Z4 Canada; ^2^ Biodiversity Research Centre University of British Columbia Vancouver BC V6T 1Z4 Canada; ^3^ Biosciences University of Exeter Penryn Campus Penryn TR10 9FE UK

**Keywords:** Between‐sex genetic correlation, genetic architecture, knockout, rFM, sexual dimorphism

## Abstract

The majority of the genome is shared between the sexes, and it is expected that the genetic architecture of most traits is shared as well. This common architecture has been viewed as a major source of constraint on the evolution of sexual dimorphism (SD). SD is nonetheless common in nature, leading to assumptions that it results from differential regulation of shared genetic architecture. Here, we study the effect of thousands of gene knockout mutations on 202 mouse phenotypes to explore how regulatory variation affects SD. We show that many traits are dimorphic to some extent, and that a surprising proportion of knockouts have sex‐specific phenotypic effects. Many traits, regardless whether they are monomorphic or dimorphic, harbor cryptic differences in genetic architecture between the sexes, resulting in sexually discordant phenotypic effects from sexually concordant regulatory changes. This provides an alternative route to dimorphism through sex‐specific genetic architecture, rather than differential regulation of shared architecture.

In organisms with separate sexes, different evolutionary interests of males and females can lead to divergent trait optima, which can be realized through the evolution of sexual dimorphism (SD). The change from monomorphic to dimorphic requires that the underlying genetic mechanisms be decoupled between males and females. However, even in species with sex chromosomes, males and females share the vast majority of their genome (Bachtrog et al. 2014), leading to the expectation that traits are controlled by the same loci in both sexes (Lande 1980). This shared genomic architecture is typically considered a source of significant constraint on the evolution of dimorphism (Stewart and Rice 2018), as traits would need to first become genetically decoupled between females and males before divergence can occur (Lande 1980; Poissant et al. 2010; Hermansen et al. 2018). Shared trait architecture can lead to intralocus sexual conflict (Rice and Chippindale 2001), where alleles at a locus have different fitness effects in males and females, and is this assumed to limit the degree to which the sexes can achieve their respective fitness optima (Hansen 2006). Indeed, the constraints on the evolution of SD are often considered both pervasive and persistent, resulting in enduring sexually conflict for many traits (Rice and Chippindale 2001; Chenoweth et al. 2008; Poissant et al. 2010; Ruzicka et al. 2019). This persistent constraint is, however, difficult to reconcile with the fact that SD evolves rapidly (Stewart and Rice 2018), is seen in a broad array of traits, and differs markedly among related species (Owens and Hartley 1998).

It has been suggested that SD arises from regulatory differences between males and females (Ellegren and Parsch 2007; Mank 2017), and there are good examples of this (e.g., Galouzis and Prud'homme 2021). Indeed, recent genome‐wide scans in fruit flies have shown that protein coding sequence differences are overrepresented among evolutionarily persistent variants thought to be maintained by sexual antagonism (Ruzicka et al. 2019). This might suggest that conflict over coding sequence variation is much harder to resolve compared to conflict over gene expression. However, functional studies have revealed that the genes underlying some dimorphisms are not expressed differently between the sexes (Khila et al. 2012). This indicates that sex‐biased expression alone cannot explain all dimorphism, and other mechanisms may exist.

Another perspective on the genetics of sexually dimorphic traits stems from investigations grounded in quantitative genetic theory (Lande 1980). By comparing the phenotypes of individuals of known relatedness, usually through breeding designs or pedigrees, one can estimate the between‐sex genetic correlation ($r_{\mathrm{fm}}$) for a trait of interest. This correlation describes the extent to which a particular genotype affects both male and female phenotypes in the same way. If $r_{\mathrm{fm}}\approx 1$, genotypes affect males and females similarly (i.e., brothers and sisters look alike), whereas if $r_{\mathrm{fm}}\approx 0$, male and female phenotypes vary independently (Lande 1980). This estimate of $r_{\mathrm{fm}}$ is based on autosomal additive standing genetic variation and measures the additive effects of the many genetic variants that exist in that population at that time. It can therefore be used to predict the extent to which a population can respond to sexually divergent selection. Because this $r_{\mathrm{fm}}$ estimate is based on the additive genetic variance, we will denote it here as $r_{\mathrm{fm}}^{A}$ for clarity.

Average estimates of $r_{\mathrm{fm}}^{A}$ often approach 1 (Poissant et al. 2010), suggesting that there is little standing genetic variation with sex‐specific effects. However, these estimates are also interpreted by many to reflect the extent to which the autosomal genetic architecture underlying the trait is shared between the sexes (Chenoweth et al. 2008; Poissant et al. 2010; Griffin et al. 2013; e.g., Stewart and Rice 2018). In other words, a strongly positive $r_{\mathrm{fm}}^{A}$ is interpreted to mean that the gene network that produces the phenotypic trait value is largely identical between the sexes, suggesting that genetic architecture needs to be decoupled before SD can evolve. Furthermore, if $r_{\mathrm{fm}}^{A}$ is an evolutionary important constraint, one would expect those traits with weak $r_{\mathrm{fm}}^{A}$ to be more likely to evolve SD, resulting in a negative relationship (Bonduriansky and Rowe 2005; Fairbairn and Roff 2006; Poissant et al. 2010). Alternatively, selection in favor of SD may drive reductions in $r_{\mathrm{fm}}^{A}$, leading to the same prediction. This negative association is supported by the prevailing evidence (Poissant et al. 2010); however, the correlation varies widely between studies, and $r_{\mathrm{fm}}^{A}$ is generally a poor predictor of SD. Furthermore, $r_{\mathrm{fm}}^{A}$ has been shown to be quickly eroded under artificial selection (Delph et al. 2011), suggesting that strong genetic correlations need not translate into significant evolutionary constraints.

$r_{\mathrm{fm}}^{A}$ estimates provide a statistical description of genotype to phenotype mapping across the sexes and are an aggregate across standing genetic variation in the population; however, we know very little about the loci that underlie this statistic. Additionally, this metric does not reveal whether sexually discordant phenotypic effects are more often the product of variation in protein coding sequence or expression. Here, we use high‐throughput phenotype data from a genome‐wide panel of gene knockouts in mice to reveal unexpected differences in the gene expression architecture between the sexes (The International Mouse Phenotyping Consortium et al. 2016; Karp et al. 2017). We find that although most phenotypic traits are dimorphic, even many monomorphic traits harbor sex‐dependent architectures, indicating substantial cryptic sex‐specific variation. Changes in both sexes to these loci through expression may provide a way for SD to rapidly evolve, as traits are already partially decoupled and the phenotypic effect differs between males and females. These findings imply that the evolutionary constraint in SD may be more easily overcome than previously thought and explain the broad diversity of SD observed in nature, as well as the apparent rapid evolution of many sexually dimorphic traits.

## Methods

We evaluated the sex‐specific effects of gene expression change by leveraging data from large‐scale high‐throughput phenotyping of gene knockout lines from the International Mouse Phenotyping Consortium (IMPC) (The International Mouse Phenotyping Consortium et al. 2016). The IMPC uses highly standardized phenotyping assays on C57BL/6 inbred mice. Both control mice and phenotype knockout lines are tested continuously, with the eventual goal of knocking out each gene in the mouse genome. This immense scientific effort provides an unprecedented opportunity to quantify the between‐sex genetic correlation across many traits and many genotypes in highly standardized conditions.

We selected phenotypes for analysis by requesting all unidimensional continuous traits, excluding legacy pipelines. We also excluded traits that were not measured in both sexes, fitness‐related traits (such as reproductive screening), body size (we analyzed body size separately), traits with fewer than 100 genotypes, and traits that were clearly not actually continuous (such as a count of the number of ribs). After triage, we had 260 traits for which we downloaded all available phenotype data, including both knockout phenotypes and control data. On average, we obtained data for 8069 control mice and 21,513 mice from 1713 knockout lines, per trait. Per knockout line, seven females and seven males were typically phenotyped.

### SD AND $r_{\mathrm{fm}}^{K}$ OF MOUSE TRAITS

If males and females share the genetic architecture of traits, knockouts should affect the phenotype of both sexes similarly, and as architectures diverge the knockout effects should diverge as well. This null model is similar to that proposed by Stewart and Rice (2018). We estimated the genetic correlation between males and females analogous to the conventional approach outlined above ($r_{\mathrm{fm}}^{A}$). However, to delineate the knockout lines from the traditional approach, we denote these estimates as $r_{\mathrm{fm}}^{K}$, where **K** denotes the genetic variance‐covariance matrix between knockout genotypes (Fig. S1). Note that $r_{\mathrm{fm}}^{K}$ measures the correlation between the phenotypic effects of genetic knockouts, whereas $r_{\mathrm{fm}}^{A}$ measures the correlation for genome‐wide additive genetic variance.

As we were interested in estimating a single value for $r_{\mathrm{fm}}^{K}$ per trait, we collapsed different sources of genetic variance into genotypes. As some gene knockouts were performed in different genetic backgrounds, some genes had multiple allelic knockouts, and some were tested in different zygosities, we defined each unique gene:allele:background:zygosity combination as a separate genotype. Note that the genetic backgrounds are all C57BL/6 mice, but a different sub‐strain.

To each of the trait datasets, we fitted a Bayesian linear mixed model with the goal of estimating both the between‐sex genetic correlation ($r_{\mathrm{fm}}^{K}$) and SD. The Bayesian approach allowed us to evaluate and propagate the uncertainty in the estimates of $r_{\mathrm{fm}}^{K}$ and SD in downstream analyses. This is especially important for $r_{\mathrm{fm}}^{K}$, because this correlation can be biased toward 0 if it is difficult to estimate (Griffin et al. 2013). We opted for the analysis of single traits as opposed to multivariate models, because phenotypes have been measured across differing sets of individuals and knockouts. Additionally, the univariate models were computationally expensive, with each model taking several days to a week to fit, and multivariate models would be logistically unfeasible. Each model had one of the phenotypes as the dependent variable, which was standardized (centered and scaled to unit variance) and transformed (see below). We included sex as a population‐level effect (also called fixed effect), allowing an average level of dimorphism across genotypes, although we did not directly use this parameter as our measurement of SD (see below). We also included body mass as a population‐level parameter, because mice are size dimorphic. Body mass was standardized (centered and scaled to unit variance) prior to analysis. All analyses were repeated without body mass, and the qualitatively similar results can be found in the Supporting Information.

To estimate $r_{\mathrm{fm}}^{K}$, we added group‐level parameters (also called random effects) of genotype for each sex, and their correlation. Finally, we added group‐level intercepts for known sources of variation when they were present, which were (1) the phenotyping center in which testing was performed, a parameter encoding several methodological differences (“meta group”), and (2) the date of testing. This leads to the final model definition in *lme4/brms* syntax: *phenotype ∼ weight + sex + (0 + sex | genotype) + (1 | center) + (1 | meta_group) + (1 | date)*. In mathematical notation, following Gelman and Hill (2006):

$$\begin{array}{ccc} & & trait_{i}∼N\left(\alpha_{j\left[i\right],k\left[i\right],l\left[i\right],m\left[i\right]}+\beta_{sex,j\left[i\right]}\left(\text{sex}\right)+\beta_{3}\left(\text{body}\;\text{mass}\right),\sigma^{2}\right),\\ & & \left(\begin{array}{c} \beta_{\text{female},j}\\ \beta_{\text{male},j} \end{array}\right)∼N\left(\left(\begin{array}{c} \mu_{\beta_{f\text{emale},j}}\\ \mu_{\beta_{\text{male},j}} \end{array}\right),\left(\begin{array}{cc} \sigma_{\beta_{\text{female},j}}^{2} & \rho_{\beta_{\text{female},j}\beta_{\text{male},j}}\\ \rho_{\beta_{\text{male},j}\beta_{\text{female},j}} & \sigma_{\beta_{\text{male},j}}^{2} \end{array}\right)\right),\\ & & \text{for}\;\text{genotype}j=1,\;\ldots ,\;J,\\ & & \alpha_{k}∼N\left(\mu_{\alpha_{k}},\sigma_{\alpha_{k}}^{2}\right),\text{for}\;\text{center}\;k=1,\ldots ,K,\\ & & \alpha_{l}∼N\left(\mu_{\alpha_{l}},\sigma_{\alpha_{l}}^{2}\right),\text{for}\;\text{meta}\;\text{group}\;l=1,\ldots ,L,\\ & & \alpha_{m}∼N\left(\mu_{\alpha_{m}},\sigma_{\alpha_{m}}^{2}\right),\text{for}\;\text{date}\;m=1,\ldots ,M. \end{array}$$

Parameter values were estimated using the *brms* (Bürkner 2017, 2018) interface to the probabilistic programming language Stan (Carpenter et al. 2017). We used weakly informative prior distributions, with priors of *N*(0, 1) for the intercept and *N*(0, 2) for the effect of body mass. For the group‐level standard deviations and residual standard deviation, we used the positive range of unit student‐*t* distributions with 5 degrees of freedom. Finally, we used a Lewandowski‐Kurowicka‐Joe (LKJ) prior with *η* = 1 for $r_{\mathrm{fm}}^{K}$, which is uniform over the range −1 to 1. Posterior distributions were obtained using Stan's no‐U‐turn HMC sampler, with two chains of 8000 iterations, with the first 4000 used as warm‐up and discarded. We additionally set the max tree depth to 20 and the adapt delta parameter to 0.9. To evaluate the ability of our models to accurately estimate the between‐sex genetic correlation, even though the sample size for each genotype was limited, we performed a simulation study (Fig. S7), confirming that our approach recovers the true value for $r_{\mathrm{fm}}^{K}$.

To satisfy the assumption of approximately normal residuals, we preceded each analysis by estimation of a Box‐Cox transformation, following the established methods by the IMPC (Kurbatova et al. 2019), using the simplified model definition: *phenotype ∼ weight + sex + (0 + sex | genotype)*. We estimated the transform using the *bcnPower* method in the *car* package (Fox et al. 2019), with model fitting performed by *lme4* (Bates et al. 2015).

After fitting all 260 trait models, we performed model criticism. For each model, we obtained the maximum $\hat{R}$ parameter, the number of divergences, and the minimum effective sample size. We removed all models that had a maximum $\hat{R}$ > 1.05, >2.5% divergent draws, or minimum effective sample size <400. Finally, we performed visual posterior predictive checks (Gabry et al. 2019), and removed models that did not reproduce the observed data distribution. Given the computational effort required for each model and that the number of successful models was more than sufficient for our analyses, we did not attempt to remedy the failing models. We visually checked to confirm that the excluded traits did not have a bias in SD or $r_{\mathrm{fm}}^{K}$. After model criticism, 202 out of 260 models remained.

For each of these models, we derived posterior distributions for three metrics of the genetic variance structure: $r_{\mathrm{fm}}^{K}$, the ratio of the sex‐specific genetic variances $\frac{V_{G(\mathrm{larger})}}{V_{G(\mathrm{smaller})}}$, and the Riemannian distance of the scaled variance‐covariance matrix from $\left[\begin{array}{cc} 1 & 1\\ 1 & 1 \end{array}\right]$. For more information about the Riemannian distance, see the Supporting Information. We then derived posterior distributions of SD by predicting average male and female phenotypes for wild‐type (i.e., control group) mice. When there were multiple genetic background variations in which a trait was tested, we used the marginal means across backgrounds. To make SD estimates comparable across traits, we used a mean standardized effect size for SD for downstream analyses, the SD index:$\frac{{\bar{x}}_{\mathrm{larger}\;\mathrm{sex}}}{{\bar{x}}_{\mathrm{smaller}\;\mathrm{sex}}}-1$. Note that the SD index requires that comparisons to 0 are biologically meaningful (i.e., traits are measured on a ratio scale), which was not true for all the traits in our dataset, such as body temperature, indices, and fractional measures. We therefore performed back transformations of the marginal means to the original scale, and we only calculated SD for 156 out of 202 traits.

After obtaining the posteriors for each trait, we used a linear model to test for a relationship between each of the three genetic (co‐)variance measures and SD. To account for uncertainty in those estimates, we performed random draws from the posterior distributions of those estimates to create 500 datasets. For each of those samples, we ran one MCMC chain of a measure *∼ SD index* model using the *brm_multiple* function, and performed inference on the combined set of 500 chains. Note that we performed a *Z*‐transformation on $r_{\mathrm{fm}}^{K}$, also called the Fisher transformation, to stabilize the variance. Additionally, we log transformed the ratio of the genetic variances and the Riemannian distance.

### DEVELOPMENT OF SIZE DIMORPHISM AND $r_{\mathrm{fm}}^{K}$

Because data on body mass were available at different ages, we analyzed this trait separately. To quantify sexual size dimorphism (SSD) during development, and associated changes in $r_{\mathrm{fm}}^{K}$, we split the body mass data into different ages. Mice were weighed once a week, with most mice being measured between 4 and 16 weeks of age. For each week, we ran the same analysis as for the separate traits outlined above.

### IDENTIFICATION OF KNOCKOUT GENOTYPES WITH SEXUALLY DISCORDANT EFFECTS

In addition to the trait‐level analyses above, we made use of the repeated phenotyping of knockouts for different traits to ascribe sexually discordant effects to particular genotypes. The concordant and discordant nature of knockout genotypes was determined by evaluating whether the genotypes were consistently ranked low or high along the concordant and discordant axes across traits. For each trait, we used the multilevel model that was used to estimate SD and $r_{\mathrm{fm}}^{K}$, described above, to obtain estimates of the male and female trait values for the measured genotypes. We extracted the posteriors for the male and female parameter for the genotype group term (Best Linear Unbiased Predictor). Note that these estimates are adjusted for body weight and environmental effects, have already undergone parameter shrinkage, and are centered around 0. We then translated the male and female phenotypes into concordant and discordant effects, by rotating the axes so that the concordant axis is the positive diagonal (female = male) and the discordant axis is the negative diagonal (female = ‐male). The absolute value along the two diagonal axes was taken, so that the effect of a genotype is larger when it is further from the population average. Because the size of the discordant effects of a genotype is strongly affected by the trait architecture (i.e., $r_{\mathrm{fm}}^{K}$), we assigned genotypes percentile ranks to aid comparison across traits.

For all genotypes that were tested on at least 100 phenotypes, we calculated the average concordant and discordant rank across traits. Credible intervals (CIs) for this average were calculated by computing that average for 100 random draws of the posteriors. We categorized genotypes as less or more discordant than average by checking whether the CI overlapped a median rank (50^th^ percentile in Fig. 4).

For the genotypes that were more discordant than average, we analyzed the gene ontology (GO) terms for the underlying knockout genes. Using *goseq* (Young et al. 2010), we tested for overrepresented GO terms, using the hypergeometric method for obtaining *P*‐values. Finally, we adjusted the *P*‐values to control the false discovery rate (Benjamini and Hochberg 1995).

### SEX‐BIASED GENE EXPRESSION AND FERTILITY

We obtained published gene expression profiles of male and female gonadal tissue from the ArrayExpress database under accession number E‐GEOD‐1148 (Rinn et al. 2004). Using *limma* (Ritchie et al. 2015), we calculated the difference in expression between the sexes (log_2_‐fold change), and empirical Bayes moderated *t*‐statistics with adjusted *P*‐values. We then classified genes as sex biased if the fold change was at least 2, and the adjusted *P*‐values were significant (*α* = 0.05). Genes that did not satisfy both those criteria were categorized as unbiased.

We then obtained female‐ and male‐specific fertility data from the IMPC (phenotypes IMPC_FER_019_001 and IMPC_FER_001_001), which are binary traits (fertile vs. infertile) where each sex has been allowed to breed with a wild‐type mate. Combining these, we defined four fertility categories: fertile, female‐limited infertile, male‐limited infertile, and infertile. To test for an association between gene expression category and fertility outcome after knockout, we performed a 3 × 4 chi‐squared test for independence.

### SOFTWARE

All analyses were performed in R version 3.6.1 (R Core Team 2019). Specific R packages used in the analyses are listed above, and the *tidyverse* (Wickham et al. 2019) was used for general data handling and visualization.

## Results

### SD AND $r_{\mathrm{fm}}^{K}$ OF MOUSE TRAITS

**Figure 1:**
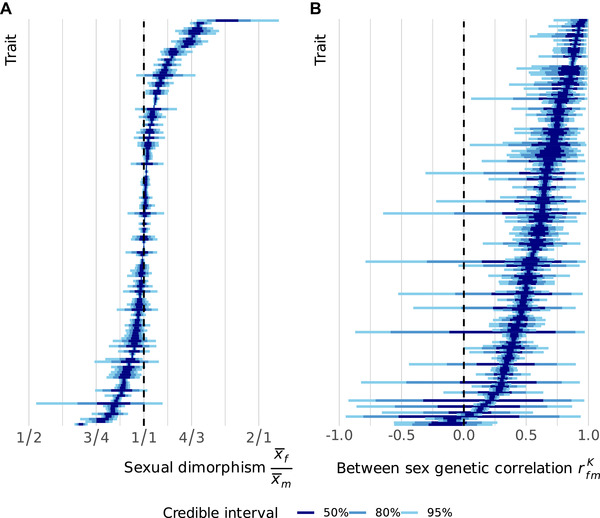
(**A**) Estimates and associated uncertainty for sexual dimorphism for each trait analyzed. Each horizontal line displays the credible intervals for one trait, where traits have been arranged by the posterior median. Shaded regions indicated the credible intervals of 50%, 80%, and 95% of the posterior densities from a multilevel model. Sexual dimorphism is averaged across the wild‐type genotypes, and defined as the ratio of female and male means. (**B**) As in panel A, but depicting the between‐sex genetic correlation $r_{\mathrm{fm}}^{K}$. Note that the traits have been arranged independently in each panel.

Many of the measured traits showed substantial SD (Fig. 1A), confirming a previous report on the IMPC data (Karp et al. 2017), with an average SD index of 0.09 [0.08, 0.10] (posterior median [95% CI]). As the large sample size in this study makes it possible to distinguish small effects with little biological relevance, we evaluated SD using equivalence testing (Wellek 2010). We compared the 95% CIs of the SD index for each trait with a region of practical equivalence (ROPE) between 0 and 0.05 (Kruschke 2018) (i.e., between 0% and 5% difference in absolute magnitude). When the entire CI falls outside the ROPE, we can be confident the sexes differ by more than 5% and the trait is considered dimorphic. We consider a trait monomorphic if we are confident there is less than a 5% difference, so when the entire CI falls within the ROPE. Under this decision rule (Kruschke 2018), dimorphic traits roughly equal monomorphic traits in number. Forty‐nine out of the 156 traits (31.4%) were found to be clearly dimorphic, whereas 47 traits (30.1%) to be monomorphic, and 60 traits (38.5%) were not classified, as their CI overlapped the 5% threshold. Some of the most monomorphic traits include calcium levels in the blood and the time spent on the periphery of an open field. Strongly dimorphic traits include a variety of immune function‐related traits, such as spleen weight and counts of different T‐cell types, as well as glucose tolerance (Table S1).

Traits showed a wide variety of estimates for $r_{\mathrm{fm}}^{K}$, from a correlation close to 1 between the phenotypes of the sexes down to correlations indistinguishable from 0 (Fig. 1B). The average correlation was clearly positive, but not as strong as we expected (0.650 [0.622, 0.689]). Surprisingly, very few traits showed a strong concordance between male and female effects, with fewer than 5% of traits having an estimate above 0.9. Some of the traits with the highest correlation are body temperature and eye morphology, whereas several immune phenotypes have a correlation close to 0 (Table S1).

**Figure 2:**
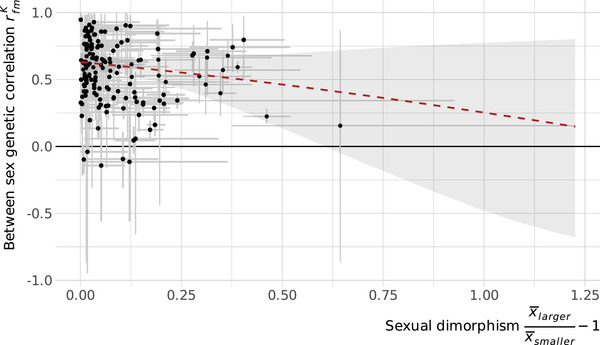
The between‐sex genetic correlation does not depend on sexual dimorphism in the trait. Each point is a trait, with error bars indicating the 95% credible interval (CI) in the estimates. The red line represents the model fit of a linear model on the Fisher‐transformed $r_{\mathrm{fm}}^{K}$, with the shaded region indicating the 95% CI, including propagation of trait level uncertainty. Sexual dimorphism is expressed as the SD ratio.

To test the constraint that high $r_{\mathrm{fm}}^{K}$ places on the evolution of dimorphism, we assessed whether $r_{\mathrm{fm}}^{K}$ is lower for more dimorphic traits, which we would expect if dimorphism is more often associated with a reduced intersexual correlation. Contrary to expectation, the between‐sex genetic correlation is not associated with SD (Fig. 2, slope: −0.49 [−1.34, 0.35]). Although there is a trend in the expected direction, the relationship is nonsignificant, and $r_{\mathrm{fm}}^{K}$ at monomorphism (i.e., the intercept) is only slightly higher than the overall average: 0.630 [0.557, 0.698].

To investigate whether there were differences in the genetic architecture of dimorphism between trait types (Poissant et al. 2010), we assigned each of the traits one of four categories: behavior, morphology, physiology, or immunity (Table S1). There is no evidence that the relationship between $r_{\mathrm{fm}}^{K}$ and SD is different for different trait categories (Fig. S2). The average $r_{\mathrm{fm}}^{K}$ of trait categories, estimated at monomorphism, can also not clearly be distinguished (Fig. S1).

Male and female genetic variances were often unbalanced, and there was a clear tendency for the male genetic variance to be larger ($\frac{V_{G(m)}}{V_{G(f)}}$ = 1.14 [1.04, 1.23]). Thus, knockout mutations have, on average, substantially larger phenotypic effects in males. It has been noted previously that mutations have larger fitness effects in male *Drosophila* (Sharp and Agrawal 2013), and differences in genetic variance between the sexes may contribute toward the evolution of dimorphism, even under a strong between‐sex genetic correlation (Wyman and Rowe 2014). However, we found no relation between the imbalance of sex‐specific variances and the level of SD (slope: 0.03 [−0.26, 0.30]). We also used a combined measure of both sex‐specific genetic variance and the between‐sex genetic correlation, the Riemannian distance to the null model (see Supporting Information), which was also not related to SD (slope: 0.75 [−0.52, 2.06], Fig. S4). Finally, we related the fraction of knockout experiments (as defined by Karp et al. 2017) with significant genotype‐by‐sex interactions to SD, which were again not related (slope: −0.01 [−0.09, 0.07], Fig. S5; see Supporting Information).

### DEVELOPMENT OF SIZE DIMORPHISM AND $r_{\mathrm{fm}}^{K}$

Body size is dimorphic in many species, including the mouse, yet it has been found numerous times that $r_{\mathrm{fm}}^{A}$ for this trait is close to 1 (Roff 2012). Nonetheless, SSD can rapidly change in response to the environment (Badyaev 2002), making this an important trait to study to better understand the link between the evolution of SD and sex‐specific architectures. As SSD is established through variable development rates and times, it is especially useful to understand when in development the effect of body size loci diverges between the sexes. Unfortunately, there are very little data available for the development of $r_{\mathrm{fm}}^{A}$, with studies usually including only two or three time points (Poissant and Coltman 2009). In contrast, the IMPC measures body weight weekly from week 3 through 16, providing the opportunity to estimate when during development the effects of expression changes become sex biased.

**Figure 3:**
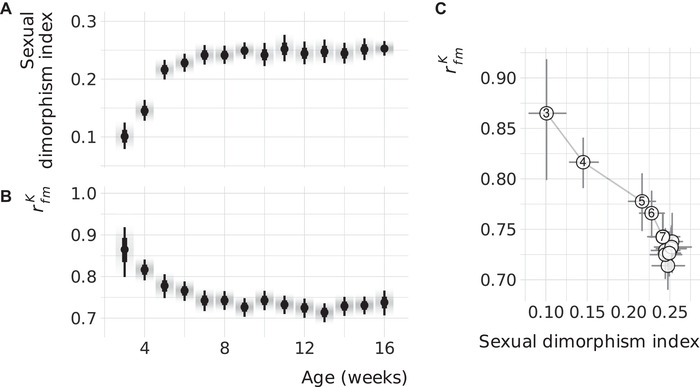
The between‐sex genetic correlation decreases as size dimorphism increases over development. (**A**) Estimates for sexual dimorphism in body mass for wild‐type mice. Points indicate the posterior median with wide and narrow line segments denoting the 66% and 95% credible intervals, respectively, and the density gradient represents the posterior density. (**B**) As in panel A, but depicting the between‐sex genetic correlation. (**C**) Association of sexual size dimorphism and the $r_{\mathrm{fm}}^{K}$ during development. Points are posterior medians with 95% credible intervals, as in panels A and B, with lines connecting subsequent week. Weeks 3 through 7 are numbered.

SSD increases strongly at the start of this period, more than doubling between weeks 3 and 7 (Fig. 3A). $r_{\mathrm{fm}}^{K}$ decreases during that same time (Fig. 3B), and both parameters stabilize around 8 weeks. The two variables follow a roughly linear negative relationship during development (Fig. 3C). A developmental link between SSD and $r_{\mathrm{fm}}^{K}$ may be the result of sexually antagonistic selection mainly acting in adulthood. This would bias sex‐specific loci to be expressed only later in development, driving an increasing SSD and decreasing $r_{\mathrm{fm}}$. Alternatively, strong trait integration during early development may pose significant constraints on the divergence of the sexes before 6 weeks.

### IDENTIFICATION OF KNOCKOUT GENOTYPES WITH SEXUALLY DISCORDANT EFFECTS

**Figure 4:**
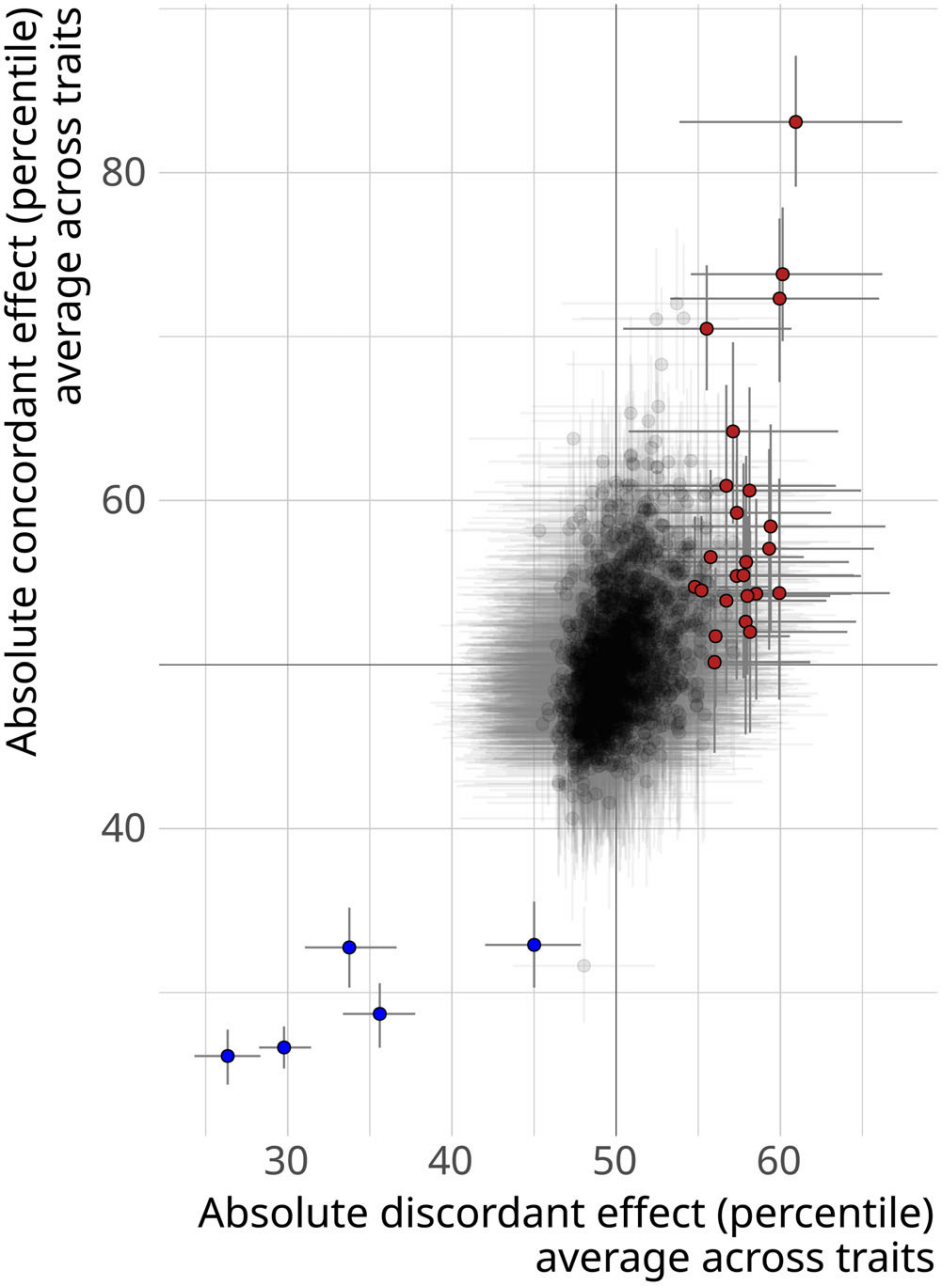
Identifying genotypes with consistent sexually discordant effects. Each point is a genotype, having been tested for at least 50 traits, with error bars denoting 95% credible intervals (CIs). The average percentile rank for the absolute sexually discordant effect of a genotype is plotted along the *x*‐axis. The *y*‐axis shows the average percentile tank for the absolute concordant effect. Red points indicate genotypes that tend to have more sexually discordant effects than other genotypes, whereas blue points are genotypes that have less discordant effects (CI does not overlap 50th percentile).

To gain insight into the extent to which sex‐specific architectures are shared between different traits, we quantified to what extent knockout genotypes have consistent sexually concordant or discordant effects. We identified five knockout genotypes that consistently had smaller sexually discordant effects, compared to other genotypes (Fig. 4). Those five genotypes also had much smaller concordant effects, indicating that their phenotypes are consistently average. Unsurprisingly, these were five wild‐type genotypes. Additionally, 24 genotypes had larger than average discordant effects (Fig. 4; Table S2). These genotypes tended to affect the sexes differently, across many traits. An analysis of Gene Ontologies for the genes that were knocked out in these genotypes revealed no significantly overrepresented categories. In contrast to the 29 discordant genotypes, 292 genotypes (out of 2543) had consistently small or large concordant effects. This difference suggests that traits are more likely to genetically covary in their average value, rather than in their dimorphism.

### SEX‐BIASED GENE EXPRESSION AND FERTILITY

Many investigations into the evolutionary significance of gene expression to SD have focused on sex‐biased gene expression (Grath and Parsch 2016). Of specific interest are expression differences in the gonads, where most sex‐biased expression occurs. In these studies, it is often assumed that gonadal expression bias reflects important sex‐specific fertility functions; however, it is usually not possible to verify this. Combining previously published gonadal expression data (Rinn et al. 2004) with fertility data from the IMPC database, however, allowed us to test whether the expression knockout of sex‐biased genes causes sex‐specific infertility.

As predicted, fertility status was significantly associated with expression bias category (i.e., male‐biased, female‐biased, or unbiased; *χ* ^2^_6_ = 76.6, *P* < 0.001; Fig. S6). Gene knockouts of female‐biased or unbiased genes led to male‐limited infertility in 1.5% of cases, but this increased to 11% of cases when knocking out male‐biased genes. Female‐limited fertility on the other hand was less common in general and showed no increase with knockouts of female‐biased genes (Fig. S6), possibly because female gametogenesis is largely encoded during fetal development and then arrested.

## Discussion

Using the extensive phenotyping effort of gene knockout mouse lines by the IMPC, we have tested for the extent of overlap in trait genetic architecture between males and females. Even in the mouse, which is relatively monomorphic when compared to many other vertebrates, it is surprisingly common for traits to show clear differences between the sexes after controlling for body size. This therefore suggests that SD is not the exception but the norm across many crucial somatic traits.

Furthermore, traits are affected differently by knockout mutations depending on the sex of the individual. This clearly illustrates that studies of gene function must account for sex, as knockout effects may only be easily detectable in one of the sexes (Karp et al. 2017; Khramtsova et al. 2019). Alterations in gene expression are often thought to be a common mechanism to resolve intralocus sexual conflict by making gene expression sex biased or sex specific (Grath and Parsch 2016). This assumes a shared genetic architecture, which is differentially regulated between the sexes. Our work suggests that the underlying architecture may differ between the sexes in many cases, and the low estimates of $r_{\mathrm{fm}}^{K}$ that we recover highlight a different potential role of gene expression in the evolution of SD.

Mutations of large regulatory effect can often be expected to alter SD, providing one way to resolve intralocus sexual conflict. However, these regulatory changes need not result in sex‐biased gene expression, as our work suggests that regulatory changes in both sexes, in this case elimination of expression in both sexes through knockouts, often predominantly only affect the phenotype of one. In other words, sexually concordant regulatory changes can result in sexually discordant phenotypic effects, and our results suggest that this commonly occurs. This provides an alternative route to dimorphism through sex‐specific genetic architecture, rather than differential regulation of shared architecture. This could, for example, be the result of interactions with sex‐biased genes in the same regulatory network, or of a sex bias in the size of the cell populations expressing the gene. It appears likely that the modulation of gene expression, either through sex bias in the downstream phenotypic effects or in the expression itself, is a major contributor to the evolution of SD.

Although mutations of large effect, especially gene deletions, can have deleterious effects on other traits through pleiotropy, most genes are nonessential (Amsterdam et al. 2004; Liao and Zhang 2007; Georgi et al. 2013). This suggests significant regulatory potential in the evolution of SD. Additionally, the knockout mutations assessed here likely represent an extreme form of regulatory variation, which we would expect to have similar, if less drastic, sex‐specific effects, and more often contribute to SD.

As others have previously indicated (Cowley and Atchley 1988; Reeve and Fairbairn 2001; Bonduriansky and Rowe 2005), $r_{\mathrm{fm}}^{A}$ may not be as strong an indicator of constraint as was originally suggested (Lande 1980). Although $r_{\mathrm{fm}}^{A}$ is very useful in describing the potential for the standing genetic variation to alter SD in a single or a few generations, it cannot detect decoupling in trait architectures that are currently lacking variation. Our results indicate that even high $r_{\mathrm{fm}}^{A}$ traits may be susceptible to changes in SD, as most traits have cryptic parts of the genetic architecture in which new mutations may have sex discordant effects. Importantly, changes in the architecture itself, such as changes in gene pathways or the recruitment of new transcription factors, are not necessary to have occurred, contrasting with a common interpretation of a strong $r_{\mathrm{fm}}^{A}$.

A potential limitation of this study is that the mice are inbred, resulting in genome‐wide homozygosity. This means that the phenotypic variation is expected to be relatively small, making the effects of knockouts appear stronger. Additionally, the effects of dominance and epistasis are artificially limited. As it has been suggested that sex‐specific dominance may be pervasive (Grieshop and Arnqvist 2018), and epistatic interactions could be affected by sex as well, our estimates of $r_{\mathrm{fm}}^{K}$ could potentially be biased upward. It is also important to note that sex‐linked genetic architecture can allow for the evolution of dimorphism. However, given the relatively small size and limited gene content of the mouse Y chromosome (Soh et al. 2014), the role of the Y in sex‐specific genetic architecture for a broad array of somatic traits is unclear.

Sex can be thought of as a hormonal context (Lawson et al. 2011; Pavličev and Cheverud 2015), and represents a form of plasticity. Many have argued that context influences phenotype through gene expression variation, and this is certainly the case for the context of sex (Mank 2017), where sex‐biased gene expression is assumed to underpin sexually dimorphic traits. Our analysis shows that sex‐dependent plasticity can arise in the absence of gene expression differences. Environment is another important context, and it has been previously noted that environment and condition can affect the degree of SD (Bonduriansky and Rowe 2005). This is evident in the IMPC data, as the degree of dimorphism varied for many knockouts based on the phenotyping center (Karp et al. 2017), although it is not clear whether there is any systematic pattern to this.

The vast majority of genotypes were neither strongly nor weakly discordant across traits, suggesting there are very few or no “sex‐specific genes” or “SD genes” but rather many different genes have sex‐specific effects on different traits. The few genotypes that did show some consistently discordant effects had no functional categories in common, also suggesting that SD is regulated differently in different traits. As we identified more genotypes that had consistently large concordant effects, the genetic covariance between trait means is likely stronger than between SD of different traits. Large‐scale analyses in a multivariate framework are needed to fully clarify the covariance of expression variance across traits and sex, to come to a complete understanding of the evolutionary constraints on SD.

In conclusion, using a dataset of unprecedented size, we demonstrated that both dimorphic and monomorphic traits harbor a surprising amount of sex‐specific genetic architecture, as sexes respond variably to knockout mutations. These results may help explain why SD is common, evolvable, and variable. Although these differences clearly indicate that the genotype‐to‐phenotype mapping is sex dependent for most traits, it remains unclear what underlying mechanisms are the cause for this. We hope future work will help elucidate proximate causes and evolutionary consequences of this work.

## AUTHOR CONTRIBUTIONS

Both authors conceived of the study, WvdB performed the data analysis, and both authors wrote the manuscript.

## COMPETING INTEREST

The authors declare no competing interest.

## DATA ARCHIVING

No new data were collected for this study. All raw phenotype data are available from the International Mouse Phenotyping Consortium (https://www.mousephenotype.org/). The gene expression profiles of male and female gonadal tissue are available from the ArrayExpress database under accession number E‐GEOD‐1148. All estimates used in downstream analyses are available in the Supporting Information.

Associate Editor: S. Wright

## Supporting information




**Figure S1**: Analysis of sex‐specific genetic variance between IMPC knock‐out lines, using spleen weight as an example.
**Figure S2**: Relationship between sexual dimorphism and the between sex genetic correlation *r^K^_mf_*.
**Figure S3**: Comparison of *r^K^_mf_* at monomorphism (the model intercept) between trait categories.
**Figure S4**: The Riemannian distance to the null model (see Supplementary Methods) does not depend on sexual dimorphism in the trait.
**Figure S5**: The fraction of knock‐out experiments with significant sex‐by‐genotype interaction does not depend on sexual dimorphism in the trait.
**Figure S6**: Fertility of gene knock‐out lines, comparing between genes that have sex‐biased expression in the gonads, and genes that have unbiased expression.
**Figure S7**: As Figure 1, but without accounting for body mass.
**Figure S8**: As Figure 2, but without accounting for body mass.
**Figure S9**: A simulation study on the potential for biased estimates for *r^K^_mf_* from our modelling approach.
**Figure S10**: Comparison between our estimates of *r^K^_mf_* and the fraction of significant sex‐by‐genotype interactions reported by Karp *et al*.
**Table S1**: Estimated parameters for each trait.
**Table S2**: List of genotypes with consistently low or consistently high discordant ranks.
