# Supplementary material for: Widespread cryptic variation in genetic architecture between the sexes
Source: Evol Lett. 2021 Jul 3;5(4):359–69. doi: 10.1002/evl3.245 (PMC8327960; doi:10.1002/evl3.245)
Supplement: Supplementary file 1 — Figure S1: Analysis of sex‐specific genetic variance between IMPC knock‐out lines, using spleen weight as an example. Figure S2: Relationship between sexual dimorphism and the between sex genetic correlation rK mf . Figure S3: Comparison of rK mf at monomorphism (the model intercept) between trait categories. Figure S4: The Riemannian distance to the null model (see Supplementary Methods) does not depend on sexual dimorphism in the trait. Figure S5: The fraction of knock‐out experiments with significant sex‐by‐genotype interaction does not depend on sexual dimorphism in the trait. Figure S6: Fertility of gene knock‐out lines, comparing between genes that have sex‐biased expression in the gonads, and genes that have unbiased expression. Figure S7: As Figure 1, but without accounting for body mass. Figure S8: As Figure 2, but without accounting for body mass. Figure S9: A simulation study on the potential for biased estimates for rK mf from our modelling approach. Figure S10: Comparison between our estimates of rK mf and the fraction of significant sex‐by‐genotype interactions reported by Karp et al. Table S1: Estimated parameters for each trait. Table S2: List of genotypes with consistently low or consistently high discordant ranks. [file EVL3-5-359-s001.docx]

## Supplementary Methods

### Riemannian distance

The possibility of a trait evolving away from monomorphism is affected both by the between-sex genetic correlation ($r_{fm}^{A}$) and the sex-specific genetic variance ($V_{G}$). In the main text, we discuss the relationships of sexual dimorphism to both these variables, and find that neither are clearly related. It could potentially be the case, however, that a relationship may become apparent when considering the combined influence of both factors. To this end, we calculated how different the genetic variance-covariance (VCV) matrix of each trait was to a null model. This null model is the simple case where the genetic variances are equal between the sexes, and the between sex correlation is 1. This can be represented by the VCV matrix $\left[ \begin{matrix} 1 & 1 \\ 1 & 1 \end{matrix} \right]$.

To compare the observed VCV and the null model, we computed the Riemannian distance between them. The Riemannian distance expresses how far the two matrices are apart along a geodesic on the Riemannian manifold that connects all VCV matrices (Maître & Mitteroecker, 2019). This distance will become larger, as the between-sex genetic correlation decreases, and the sex-specific genetic variances diverge. It can therefore be seen as representing the deviation of a trait from an identical genetic architecture between the sexes.

Since this distance would be affected by the scale of the observed VCV (so by the total genetic variance), we standardized both matrices by dividing them by their trace. The Riemannian distances were computed using vcvComp R package (Maître & Mitteroecker, 2019). The relationship between the SD index and the Riemannian distance metric is shown in Figure S4.

### Comparison to Karp *et al.*

Instead of estimating the quantitative genetic parameters that describe the variation among genotypes, one could instead take the simpler approach to look at whether dimorphic traits more often show significant genotype-by-sex interactions in knock-out experiments. Previous work (Karp *et al.*, 2017) has estimated both SD and the number of significant interactions, although the aims of that work were different and they did not relate the two quantities. Here we provide a simple analysis of this relationship.

Karp *et al.* did not perform trait level analyses, but rather compiled groups of experiments with matched controls to estimate SD in wildtype mice, and performed individual statistical tests for each separate knock-out experiment. We obtained their results for same traits used in our analyses, and calculated the SD index (using the reported female and male trait means), and the fraction of knock-out experiments in which there was a significant genotype-by-sex effect. We then fitted an analogous model to what we used in the main text to analyze the relationship between $r_{fm}^{K}$ and the SD index, but added trait level intercepts and slopes to account for pseudo-replication (since traits could have multiple estimates). Figure S5 shows the result of this analysis, demonstrating no relationship between the fraction of significant interactions, and the level of dimorphism in the trait.

The likelihood of obtaining a significant genotype-by-sex interaction in a knock-out experiment will depend on the genetic architecture, and we therefore expect our estimates to be related to this more simple metric. Indeed, we clearly see that as $r_{fm}^{K}$ approaches 1, the number of significant interactions becomes smaller (Figure S10). However, there is considerable variation in this relationship. This is likely the result of the dichotomization of effects as significant or non-significant, with no consideration of effect size and varying levels of power.

## Supplementary Figures


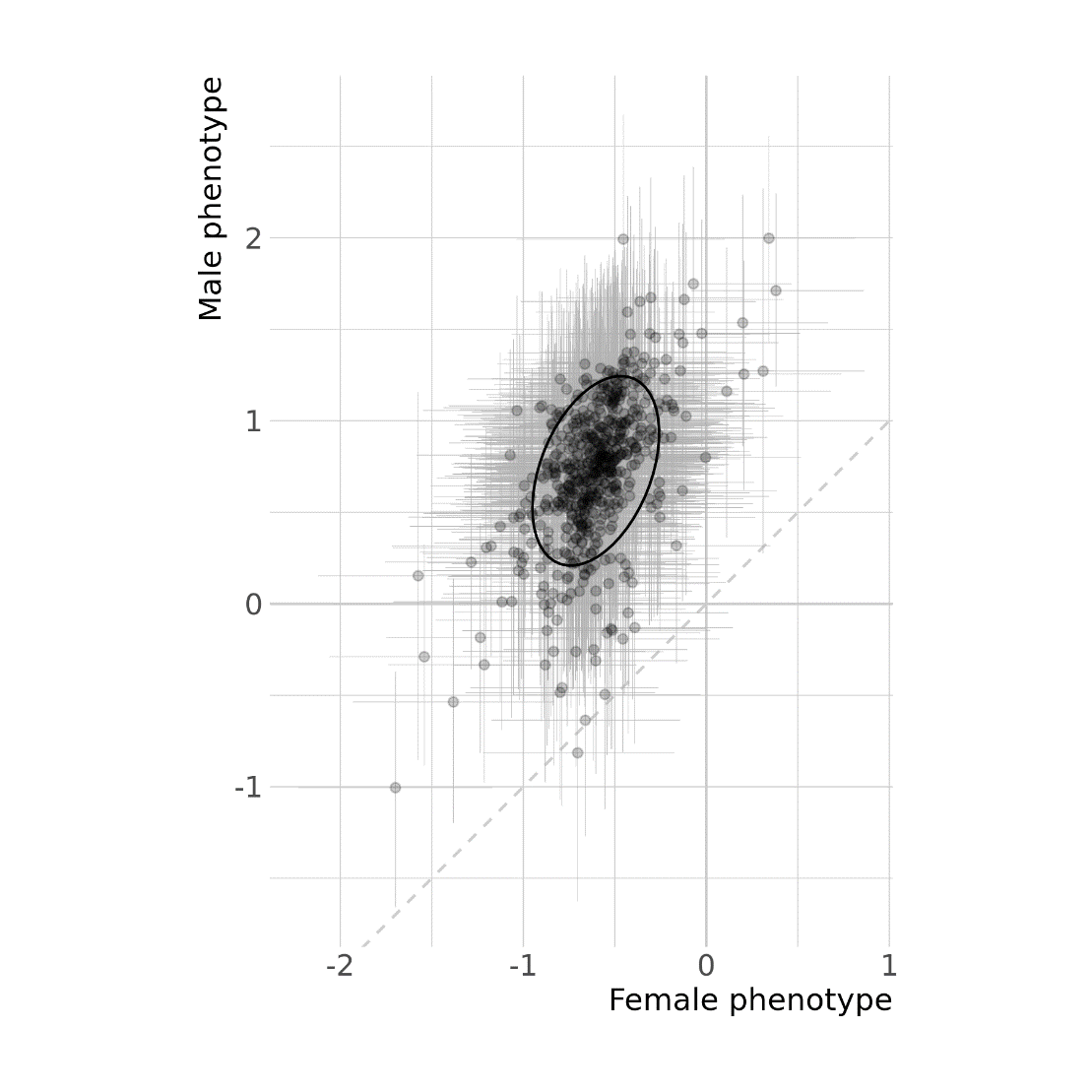


**Figure S1**: Analysis of sex-specific genetic variance between IMPC knock-out lines, using spleen weight as an example. Each point represents an estimate (best linear unbiased predictor, BLUP) of the male and female phenotypes of one knock-out genotype, estimated from a Bayesian mixed model accounting for body weight and other factors. Spleen weight was Box-Cox transformed and standardized before analysis. The ellipse drawn describes the observed genetic variance-covariance matrix, and the diagonal dotted line signifies monomorphism. $r_{fm}^{K}$ for this trait is 0.40 (posterior median, 95% credible interval: [0.23, 0.56]).


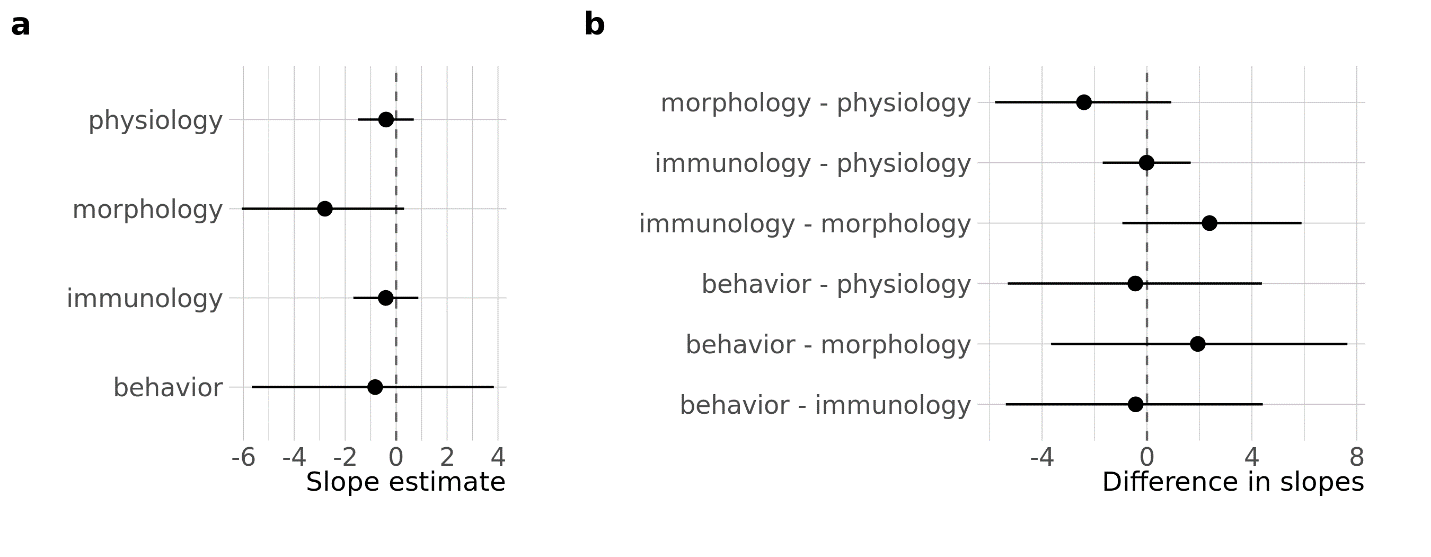


**Figure S2:** Relationship between sexual dimorphism and the between sex genetic correlation $r_{mf}^{K}$. Panel **a** shows the marginal estimates for the slope (${Zr}_{mf}^{K}\sim SD$) for each trait category. Panel **b** shows the contrasts between the slopes.


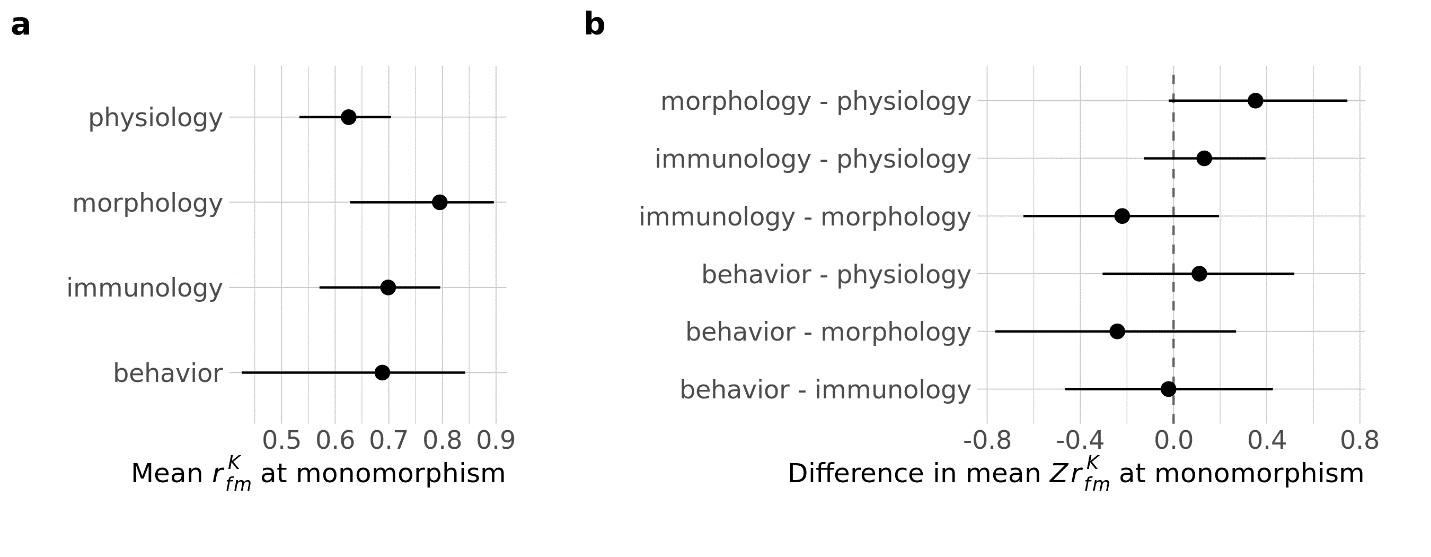


**Figure S3:** Comparison of $r_{mf}^{K}$ at monomorphism (the model intercept) between trait categories. Panel **a** shows the marginal means. Panel **b** shows the contrasts between the means.


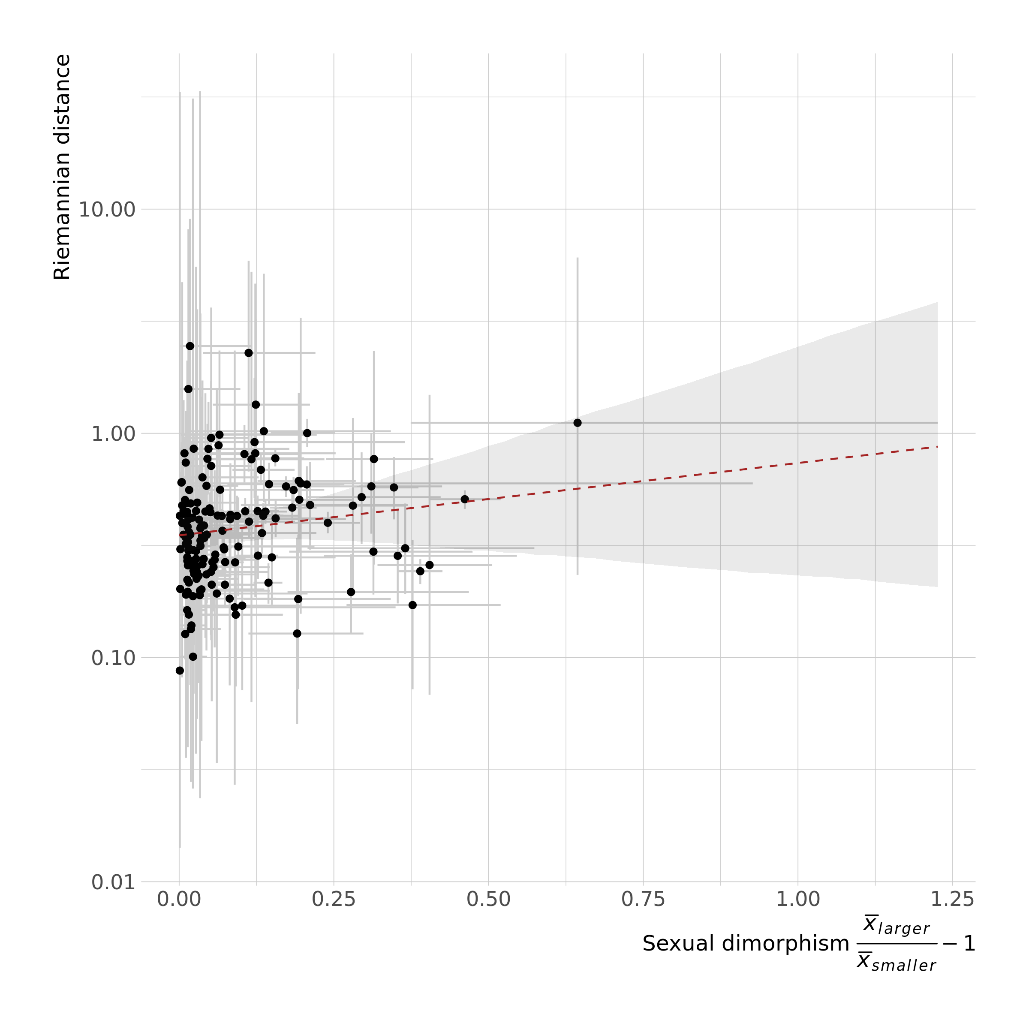


**Figure S4:** The Riemannian distance to the null model (see Supplementary Methods) does not depend on sexual dimorphism in the trait. Each point is a trait, with error bars indicating the 95% credible interval (CI) in the estimates. The red line represents the model fit of a linear model on the log-transformed Riemannian distance, with the shaded region indicating the 95% credible interval, including propagation of trait level uncertainty. Sexual dimorphism is expressed as the SD ratio.


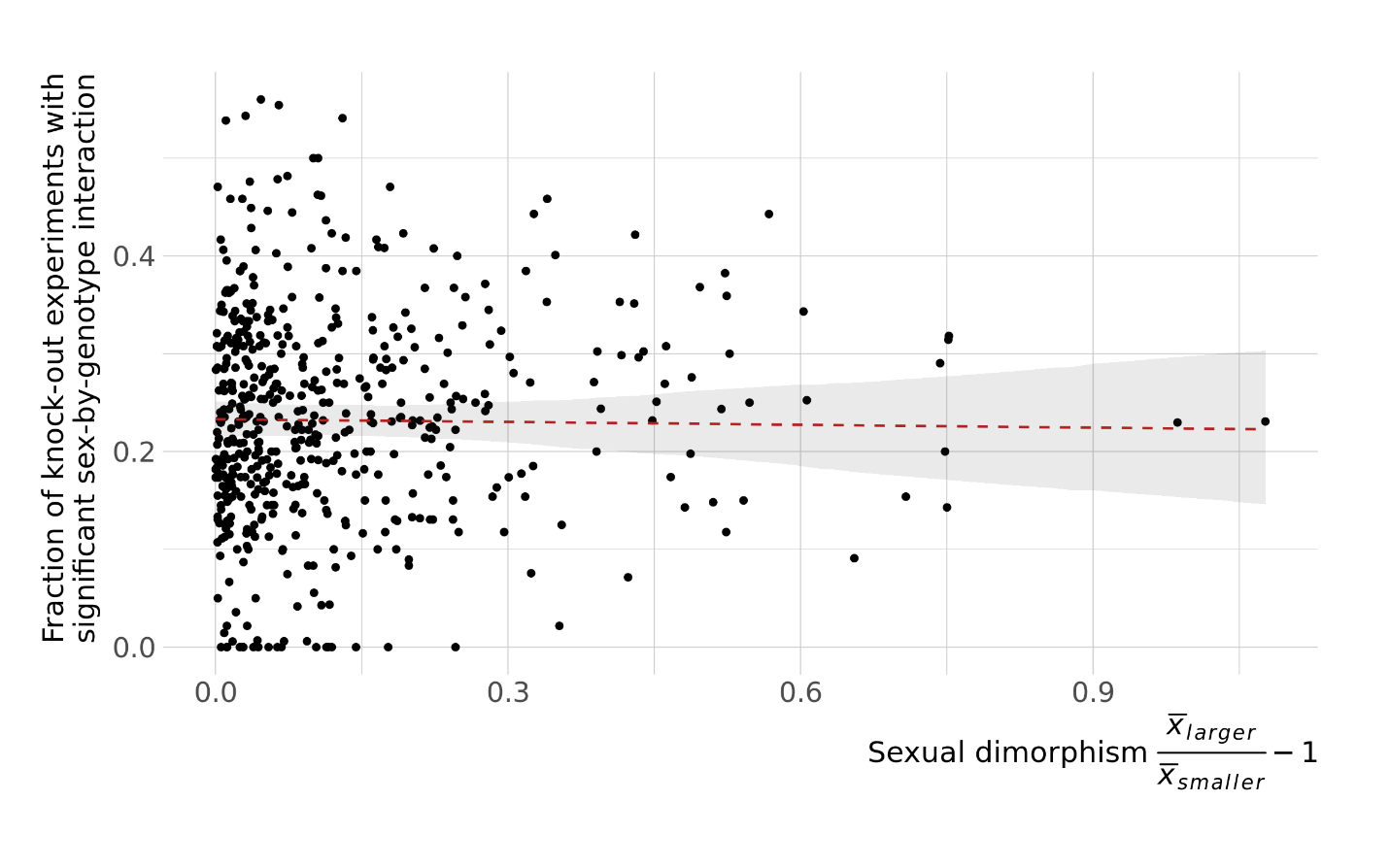


**Figure S5:** The fraction of knock-out experiments with significant sex-by-genotype interaction does not depend on sexual dimorphism in the trait. Each point is an experimental group, as defined by (Karp *et al.*, 2017). The red line represents the model fit of a linear model including trait level intercepts and slopes, with the shaded region indicating the 95% credible interval. Sexual dimorphism is expressed as the SD ratio.


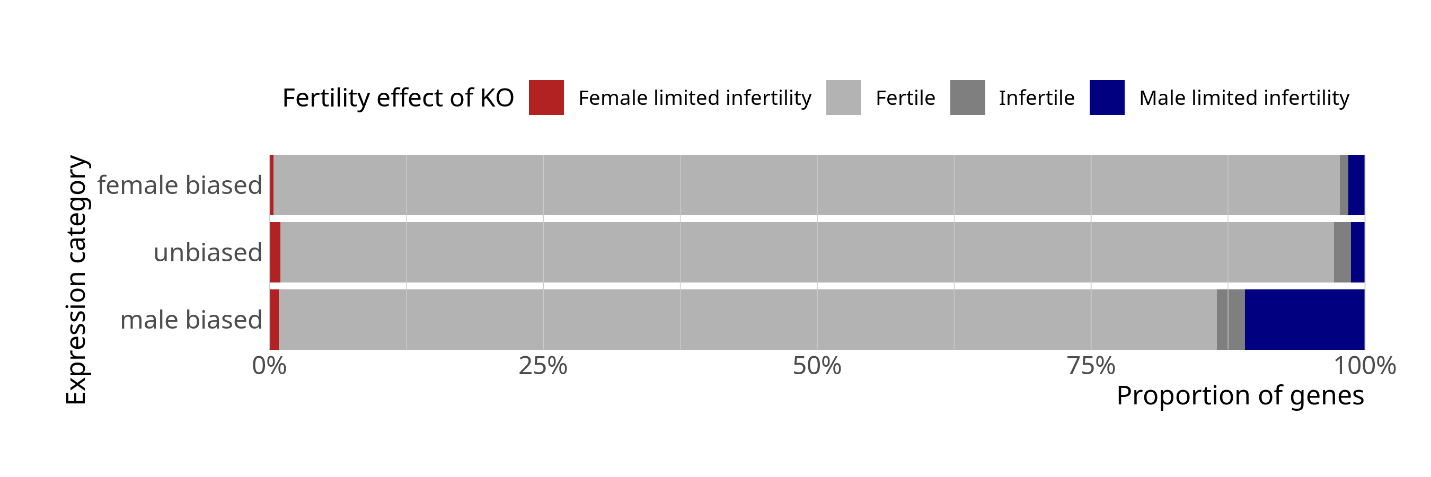


**Figure S6:** Fertility of gene knock-out lines, comparing between genes that have sex-biased expression in the gonads, and genes that have unbiased expression.


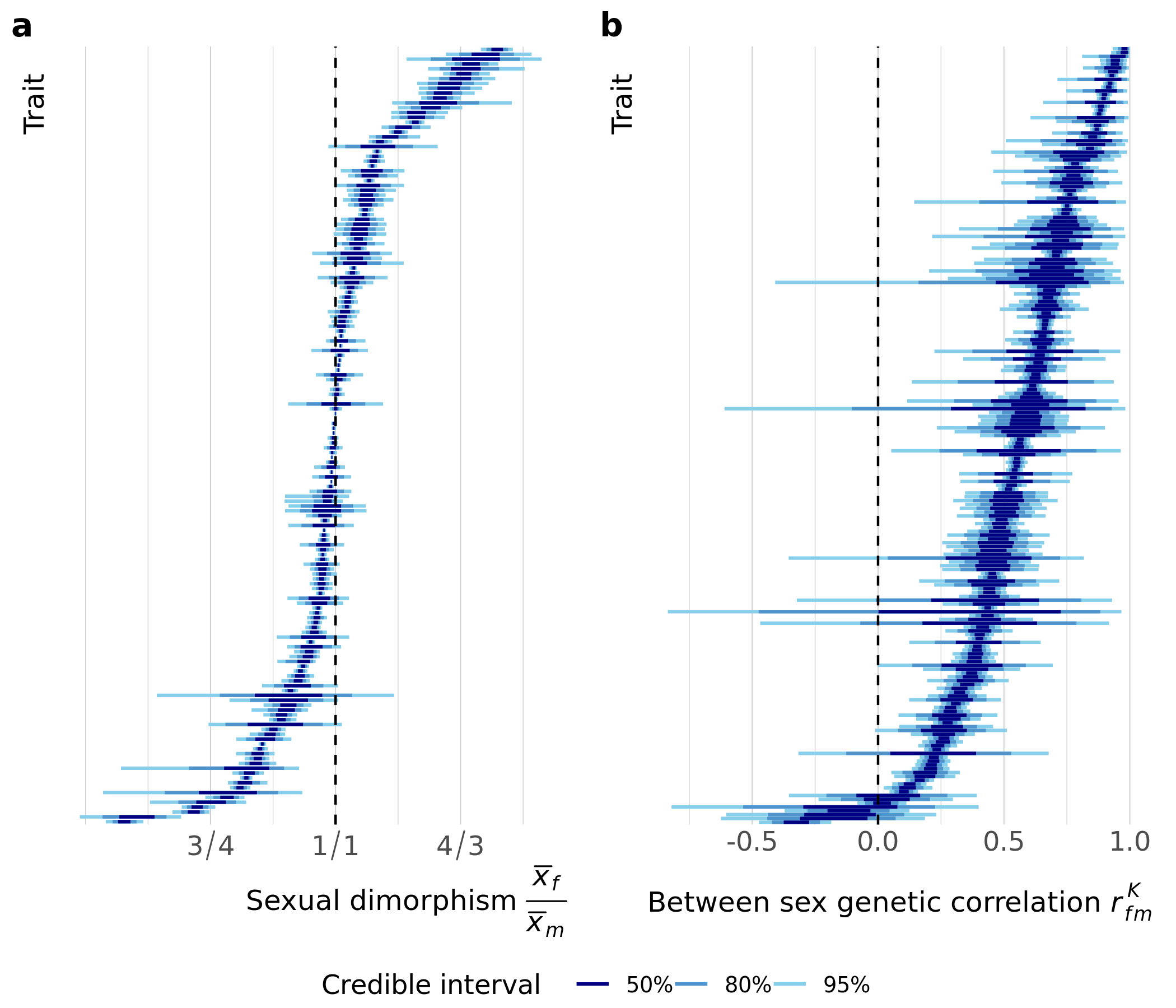


**Figure S7:** As Figure 1, but without accounting for body mass. (**a**) Estimates and associated uncertainty for sexual dimorphism for each trait analyzed. Each line displays the credible intervals for one trait, where traits have been arranged by the posterior median. Shaded regions indicated the credible intervals of 50%, 80% and 95% of the posterior densities from a multi-level model. Sexual dimorphism is averaged across the wild-type genotypes, and defined as the ratio of female and male means. (**b**) As in (a), but depicting the between sex genetic correlation $r_{fm}^{K}$. Note that the traits have been arranged independently in each panel.

**
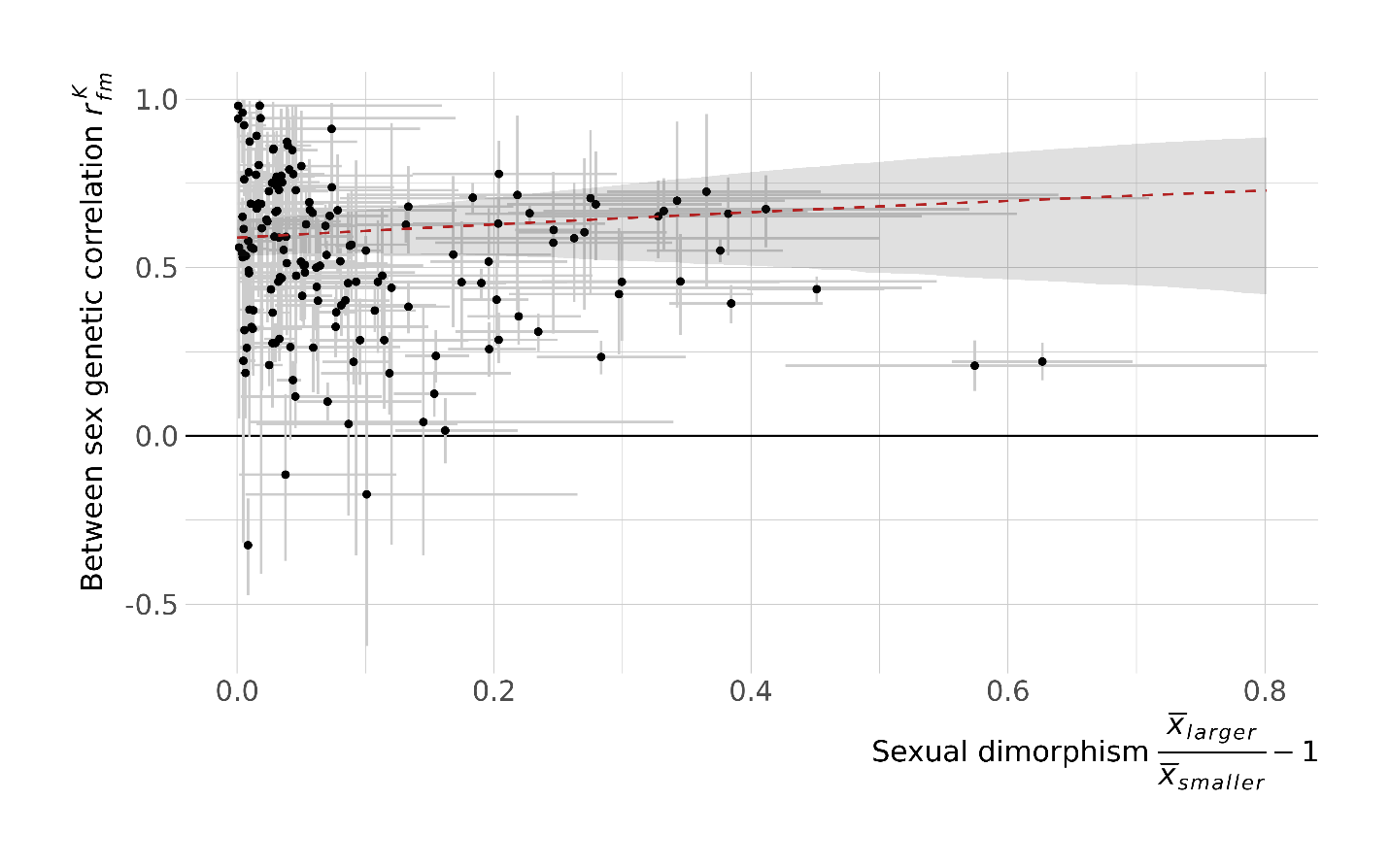
**

**Figure S8:** As Figure 2, but without accounting for body mass. The between sexual genetic correlation does not depend on sexual dimorphism in the trait. Each point is a trait, with error bars indicating the 95% credible interval (CI) in the estimates. The line represents the model fit of a linear model on the Fisher-transformed $r_{fm}^{K}$, with the shaded region indicating the 95% credible interval, including propagation of trait level uncertainty.

**
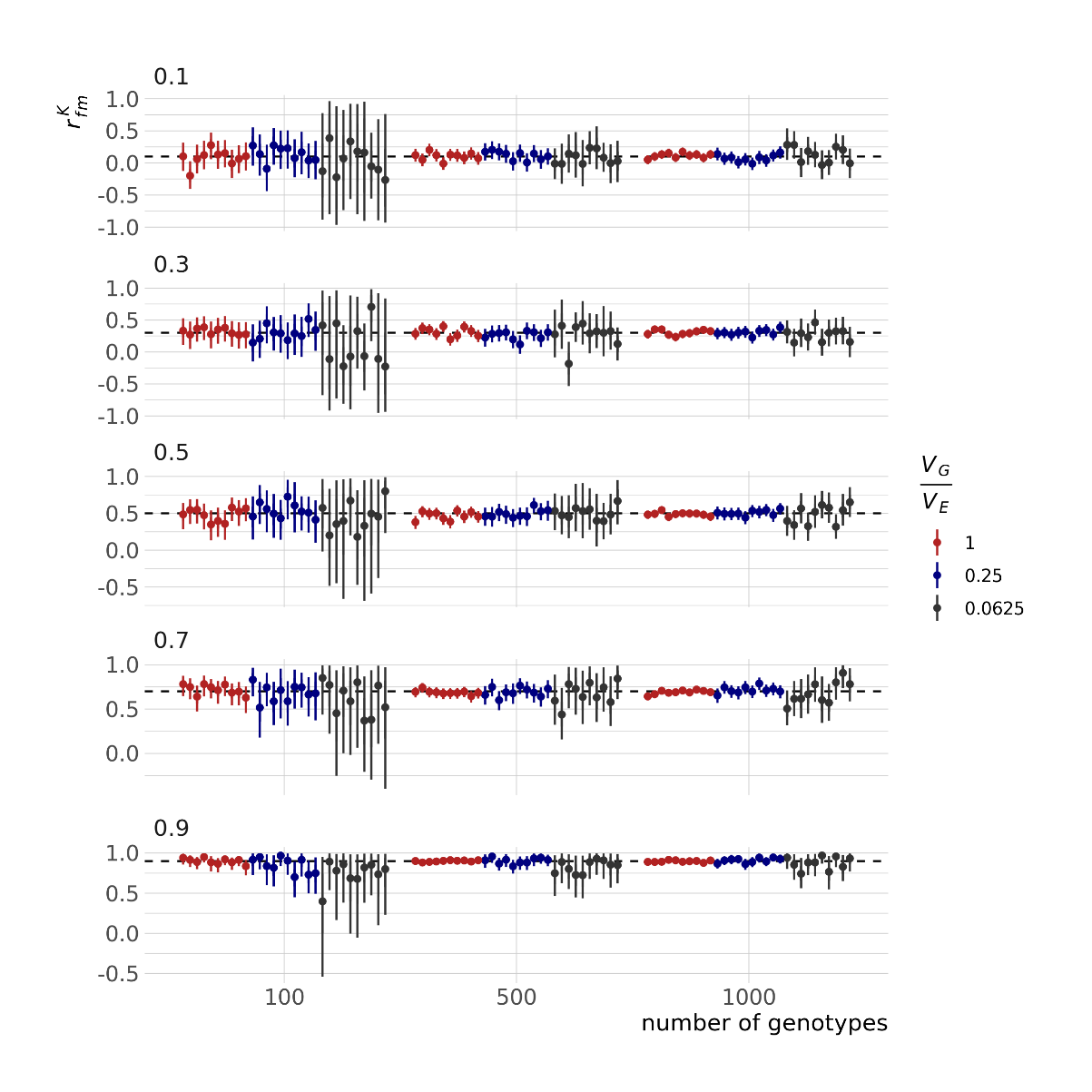
**

**Figure S9:** A simulation study on the potential for biased estimates for $r_{fm}^{K}$ from our modelling approach. We simulated data, with varying number of genotypes available (100, 500 or 1000), varying true values for $r_{fm}^{K}$ (0.1, 0.3, 0.5, 0.7, 0.9), and varying proportions of genetic variance (compared to non-genetic variance, 1:1, 1:4, 1:16). We replicated the limited data available for each genotype in the IMPC data, and simulated 7 females and 7 males per genotype. The figure shows the point estimate and 95% Credible Interval for 10 fitted models for each scenario.

**
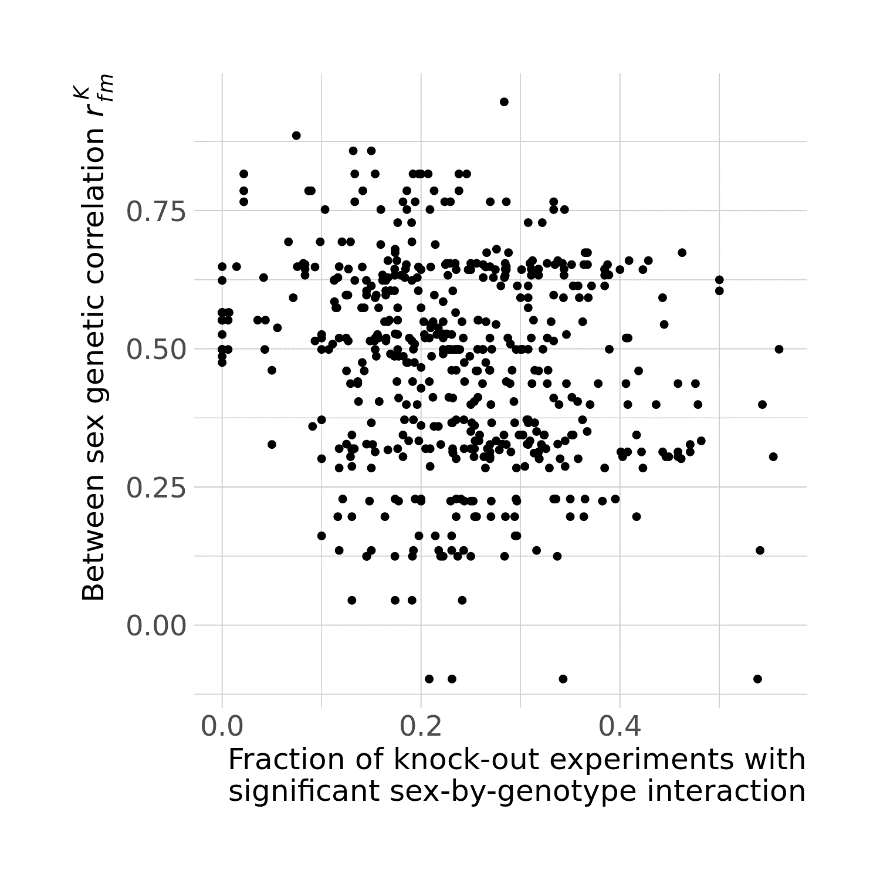
**

**Figure S10:** Comparison between our estimates of $r_{fm}^{K}$ and the fraction of significant sex-by-genotype interactions reported by Karp *et al.* Note that points do not represent traits (see Supplementary Methods).

## Supplementary Tables

**Table S1**: Estimated parameters for each trait. *n* denotes the total sample size (number of individuals tested), *genotypes* the number of different genotypes, *SD* is sexual dimorphism, *rFM* is the between sex genetic correlation. *SD* and *rFM* are given as the estimate with 95% Credible Intervals.

| Trait ID | Trait name | Trait category | n | genotypes | SD index | rFM |
| --- | --- | --- | --- | --- | --- | --- |
| IMPC_ACS_001_001 | Response amplitude - BN | physiology | 68729 | 3611 |  | 0.748 [0.673, 0.829] |
| IMPC_ACS_002_001 | Response amplitude - PP1 | physiology | 68731 | 3611 |  | 0.765 [0.704, 0.827] |
| IMPC_ACS_003_001 | Response amplitude - PP2 | physiology | 68725 | 3611 |  | 0.747 [0.679, 0.811] |
| IMPC_ACS_004_001 | Response amplitude - PP3 | physiology | 68731 | 3611 |  | 0.749 [0.698, 0.804] |
| IMPC_ACS_005_001 | Response amplitude - PP4 | physiology | 31535 | 1302 |  | 0.882 [0.812, 0.947] |
| IMPC_ACS_006_001 | Response amplitude - S | physiology | 68343 | 3594 |  | 0.872 [0.842, 0.9] |
| IMPC_ACS_007_001 | Response amplitude - PP1_S | physiology | 68350 | 3594 |  | 0.879 [0.852, 0.907] |
| IMPC_ACS_008_001 | Response amplitude - PP2_S | physiology | 68344 | 3594 |  | 0.89 [0.862, 0.917] |
| IMPC_ACS_009_001 | Response amplitude - PP3_S | physiology | 68350 | 3594 |  | 0.871 [0.842, 0.898] |
| IMPC_ACS_010_001 | Response amplitude - PP4_S | physiology | 31535 | 1302 |  | 0.856 [0.809, 0.906] |
| IMPC_ACS_033_001 | % Pre-pulse inhibition - PPI1 | physiology | 68333 | 3594 | 0.044 [0, 0.107] | 0.786 [0.635, 0.944] |
| IMPC_ACS_034_001 | % Pre-pulse inhibition - PPI2 | physiology | 68327 | 3594 | 0.022 [0, 0.042] | 0.817 [0.757, 0.876] |
| IMPC_ACS_035_001 | % Pre-pulse inhibition - PPI3 | physiology | 68333 | 3594 | 0.01 [0, 0.023] | 0.766 [0.715, 0.818] |
| IMPC_ACS_036_001 | % Pre-pulse inhibition - PPI4 | physiology | 31536 | 1302 | 0.02 [0, 0.039] | 0.752 [0.673, 0.835] |
| IMPC_ACS_037_001 | % Pre-pulse inhibition - Global | physiology | 31530 | 1301 | 0.016 [0, 0.046] | 0.724 [0.626, 0.81] |
| IMPC_ABR_002_001 | Click-evoked ABR threshold | physiology | 17535 | 3090 |  | 0.919 [0.891, 0.945] |
| IMPC_ABR_004_001 | 6kHz-evoked ABR Threshold | physiology | 30798 | 4404 |  | 0.904 [0.869, 0.937] |
| IMPC_ABR_006_001 | 12kHz-evoked ABR Threshold | physiology | 30806 | 4404 |  | 0.923 [0.893, 0.952] |
| IMPC_ABR_008_001 | 18kHz-evoked ABR Threshold | physiology | 30781 | 4403 |  | 0.899 [0.869, 0.931] |
| IMPC_ABR_010_001 | 24kHz-evoked ABR Threshold | physiology | 30685 | 4400 |  | 0.879 [0.841, 0.917] |
| IMPC_ABR_012_001 | 30kHz-evoked ABR Threshold | physiology | 30185 | 4397 |  | 0.833 [0.785, 0.885] |
| IMPC_DXA_002_001 | Fat mass | morphology | 81898 | 4346 | 0.155 [0.123, 0.198] | 0.313 [0.267, 0.359] |
| IMPC_DXA_005_001 | Bone Mineral Content (excluding skull) | morphology | 81045 | 4316 | 0.069 [0.059, 0.081] | 0.305 [0.265, 0.348] |
| IMPC_DXA_006_001 | Body length | morphology | 67662 | 3724 | 0.002 [0, 0.004] | 0.499 [0.458, 0.538] |
| IMPC_DXA_010_001 | Bone Area | morphology | 81046 | 4316 | 0.034 [0.024, 0.042] | 0.437 [0.387, 0.486] |
| IMPC_CBC_001_001 | Sodium | physiology | 49468 | 2470 | 0.009 [0.005, 0.012] | -0.098 [-0.202, 0.016] |
| IMPC_CBC_002_001 | Potassium | physiology | 49249 | 2470 | 0.093 [0.076, 0.112] | 0.412 [0.318, 0.512] |
| IMPC_CBC_004_001 | Urea (Blood Urea Nitrogen - BUN) | physiology | 73486 | 2949 | 0.01 [0, 0.028] | 0.461 [0.388, 0.53] |
| IMPC_CBC_005_001 | Creatinine | physiology | 65688 | 2523 | 0.107 [0.078, 0.134] | 0.284 [0.207, 0.366] |
| IMPC_CBC_006_001 | Total protein | physiology | 73404 | 2945 | 0.005 [0, 0.009] | 0.372 [0.289, 0.456] |
| IMPC_CBC_007_001 | Albumin | physiology | 74225 | 2950 | 0.064 [0.058, 0.069] | 0.301 [0.22, 0.378] |
| IMPC_CBC_008_001 | Total bilirubin | physiology | 72729 | 2939 | 0.011 [0, 0.028] | 0.526 [0.431, 0.623] |
| IMPC_CBC_009_001 | Calcium | physiology | 73880 | 2948 | 0.001 [0, 0.003] | 0.327 [0.211, 0.43] |
| IMPC_CBC_010_001 | Phosphorus | physiology | 73250 | 2947 | 0.027 [0.015, 0.041] | 0.499 [0.417, 0.58] |
| IMPC_CBC_011_001 | Iron | physiology | 47260 | 2301 | 0.185 [0.163, 0.207] | 0.162 [0.078, 0.248] |
| IMPC_CBC_012_001 | Aspartate aminotransferase | physiology | 73624 | 2950 | 0.007 [0, 0.024] | 0.574 [0.483, 0.669] |
| IMPC_CBC_013_001 | Alanine aminotransferase | physiology | 74021 | 2950 | 0.127 [0.091, 0.161] | 0.519 [0.427, 0.613] |
| IMPC_CBC_014_001 | Alkaline phosphatase | physiology | 73703 | 2937 | 0.39 [0.353, 0.425] | 0.593 [0.549, 0.635] |
| IMPC_CBC_015_001 | Total cholesterol | physiology | 73572 | 2947 | 0.058 [0.044, 0.073] | 0.643 [0.599, 0.692] |
| IMPC_CBC_016_001 | HDL-cholesterol | physiology | 67324 | 2793 | 0.127 [0.101, 0.159] | 0.614 [0.564, 0.664] |
| IMPC_CBC_017_001 | Triglycerides | physiology | 72229 | 2933 | 0.136 [0.085, 0.193] | 0.366 [0.297, 0.429] |
| IMPC_CBC_018_001 | Glucose | physiology | 73210 | 2949 | 0.044 [0, 0.088] | 0.135 [0.067, 0.206] |
| IMPC_CBC_020_001 | Fructosamine | physiology | 31243 | 1655 | 0.049 [0.031, 0.062] | 0.35 [0.268, 0.428] |
| IMPC_CBC_021_001 | Lipase | physiology | 6620 | 264 | 0.014 [0, 0.031] | 0.886 [0.737, 0.991] |
| IMPC_CBC_022_001 | Lactate dehydrogenase | physiology | 3675 | 115 | 0.123 [0.019, 0.241] | 0.466 [-0.238, 0.955] |
| IMPC_CBC_023_001 | Alpha-amylase | physiology | 37847 | 1790 | 0.144 [0.121, 0.167] | 0.643 [0.583, 0.706] |
| IMPC_CBC_024_001 | UIBC (unsaturated iron binding capacity) | physiology | 6681 | 264 | 0.07 [0.05, 0.091] | 0.429 [0.217, 0.632] |
| IMPC_CBC_025_001 | LDL-cholesterol | physiology | 21025 | 1079 | 0.113 [0.047, 0.182] | 0.361 [0.27, 0.448] |
| IMPC_CBC_026_001 | Free fatty acids | physiology | 23343 | 1416 | 0.051 [0.008, 0.088] | 0.311 [0.192, 0.428] |
| IMPC_CBC_028_001 | Creatine kinase | physiology | 31464 | 1616 | 0.052 [0, 0.102] | 0.858 [0.585, 1] |
| IMPC_CSD_032_001 | Locomotor activity | behavior | 70051 | 3822 | 0.139 [0.108, 0.173] | 0.333 [0.267, 0.397] |
| IMPC_ECH_001_001 | End-Systolic Diameter | morphology | 5954 | 733 | 0.014 [0, 0.031] | 0.625 [0.441, 0.796] |
| IMPC_ECH_002_001 | End-Diastolic Diameter | morphology | 5954 | 733 | 0.011 [0, 0.021] | 0.634 [0.45, 0.812] |
| IMPC_ECH_003_001 | Stroke Volume | physiology | 13123 | 1014 | 0.022 [0, 0.047] | 0.538 [0.337, 0.73] |
| IMPC_ECH_004_001 | Ejection Fraction | physiology | 15275 | 1186 |  | 0.636 [0.49, 0.783] |
| IMPC_ECH_005_001 | Fractional Shortening | physiology | 19414 | 1193 |  | 0.687 [0.54, 0.832] |
| IMPC_ECH_006_001 | Cardiac Output | physiology | 13101 | 612 | 0.022 [0, 0.048] | 0.527 [0.026, 0.979] |
| IMPC_ECH_008_001 | LVIDd | physiology | 19414 | 1193 | 0.011 [0, 0.024] | 0.544 [0.408, 0.672] |
| IMPC_ECH_009_001 | LVPWd | physiology | 19415 | 1193 | 0.004 [0, 0.013] | 0.317 [0.095, 0.534] |
| IMPC_ECH_010_001 | LVAWs | physiology | 8078 | 905 | 0.014 [0, 0.033] | 0.585 [0.369, 0.8] |
| IMPC_ECH_011_001 | LVIDs | physiology | 19415 | 1193 | 0.013 [0, 0.036] | 0.605 [0.461, 0.731] |
| IMPC_ECH_012_001 | LVPWs | physiology | 15274 | 1186 | 0.009 [0, 0.021] | 0.411 [0.234, 0.584] |
| IMPC_ECH_013_001 | HR | physiology | 19406 | 789 | 0.011 [0, 0.024] | 0.509 [0.283, 0.732] |
| IMPC_ECH_014_001 | Body Temp | physiology | 3798 | 194 |  | 0.966 [0.792, 1] |
| IMPC_ECH_018_001 | Respiration Rate | physiology | 10723 | 589 | 0.031 [0, 0.075] | 0.689 [0.4, 0.935] |
| IMPC_ECG_001_001 | Number of signals | physiology | 58260 | 3272 | 0.011 [0, 0.026] | 0.451 [0.282, 0.629] |
| IMPC_ECG_002_001 | HR | physiology | 61035 | 3387 | 0.015 [0.011, 0.02] | 0.486 [0.406, 0.569] |
| IMPC_ECG_003_001 | CV | physiology | 37965 | 2288 |  | 0.5 [0.279, 0.719] |
| IMPC_ECG_004_001 | RR | physiology | 61034 | 3387 | 0.015 [0.011, 0.021] | 0.475 [0.392, 0.561] |
| IMPC_ECG_005_001 | PQ | physiology | 37178 | 2247 | 0.019 [0.005, 0.032] | 0.605 [0.395, 0.8] |
| IMPC_ECG_006_001 | PR | physiology | 61036 | 3387 | 0.015 [0.009, 0.02] | 0.597 [0.363, 0.843] |
| IMPC_ECG_007_001 | QRS | physiology | 61022 | 3387 | 0.007 [0.001, 0.013] | 0.624 [0.347, 0.913] |
| IMPC_ECG_008_001 | ST | physiology | 56467 | 3204 | 0.014 [0.01, 0.019] | 0.514 [0.3, 0.714] |
| IMPC_ECG_009_002 | QTc | physiology | 3889 | 621 | 0.005 [0.001, 0.008] | 0.635 [-0.021, 0.999] |
| IMPC_ECG_010_001 | HRV | physiology | 37178 | 2247 | 0.311 [0.21, 0.424] | 0.463 [0.23, 0.685] |
| IMPC_ECG_011_001 | QTc Dispersion | physiology | 38928 | 2315 | 0.023 [0, 0.044] | 0.694 [0.396, 0.992] |
| IMPC_ECG_012_001 | Mean SR amplitude | physiology | 37172 | 2247 | 0.134 [0.071, 0.215] | 0.405 [0.308, 0.504] |
| IMPC_ECG_013_001 | Mean R amplitude | physiology | 40058 | 2315 | 0.156 [0.076, 0.243] | 0.328 [0.224, 0.428] |
| IMPC_ECG_014_001 | rMSSD | physiology | 37176 | 2247 | 0.193 [0.12, 0.276] | 0.648 [0.403, 0.908] |
| IMPC_EYE_054_001 | Min left eye lens density | morphology | 4271 | 149 |  | 0.898 [0.774, 0.999] |
| IMPC_EYE_055_001 | Max left eye lens density | morphology | 4271 | 149 |  | 0.92 [0.785, 1] |
| IMPC_EYE_056_001 | Mean left eye lens density | morphology | 4271 | 149 |  | 0.937 [0.833, 1] |
| IMPC_EYE_057_001 | Min right eye lens density | morphology | 4242 | 149 |  | 0.883 [0.751, 0.998] |
| IMPC_EYE_058_001 | Max right eye lens density | morphology | 4242 | 149 |  | 0.719 [0.536, 0.866] |
| IMPC_EYE_059_001 | Mean right eye lens density | morphology | 4242 | 149 |  | 0.798 [0.674, 0.911] |
| IMPC_EYE_063_001 | Right inner nuclear layer | morphology | 3995 | 121 | 0.011 [0, 0.035] | 0.866 [0.608, 0.998] |
| IMPC_EYE_064_001 | Right outer nuclear layer | morphology | 3997 | 121 | 0.015 [0, 0.067] | 0.411 [-0.774, 1] |
| IMPC_EYE_068_001 | Left total retinal thickness | morphology | 8765 | 305 | 0.001 [0, 0.003] | 0.947 [0.852, 1] |
| IMPC_EYE_069_001 | Left inner nuclear layer | morphology | 4073 | 121 | 0.022 [0, 0.049] | 0.909 [0.66, 1] |
| IMPC_EYE_070_001 | Left outer nuclear layer | morphology | 4072 | 121 | 0.018 [0, 0.08] | -0.04 [-0.925, 0.96] |
| IMPC_FEA_001_001 | Conditioning Baseline Freeze Count | behavior | 1551 | 135 | 0.196 [0, 0.702] | 0.343 [-0.339, 0.95] |
| IMPC_FEA_006_001 | Conditioning Baseline Maximum Motion Index | behavior | 1551 | 108 |  | 0.655 [0.267, 0.999] |
| IMPC_FEA_007_001 | Context Freeze Count | behavior | 1551 | 135 | 0.027 [0, 0.09] | 0.866 [0.534, 1] |
| IMPC_FEA_008_001 | Context Freezing Time | behavior | 1551 | 135 | 0.034 [0, 0.12] | 0.929 [0.731, 1] |
| IMPC_FEA_012_001 | Context Maximum Motion Index | behavior | 1551 | 108 |  | 0.626 [-0.403, 1] |
| IMPC_FEA_018_001 | Cue Baseline Maximum Motion Index | behavior | 1549 | 108 |  | 0.683 [0.243, 0.998] |
| IMPC_FEA_021_001 | Cue Tone % Freezing Time | behavior | 1550 | 108 |  | 0.72 [0.39, 1] |
| IMPC_FEA_094_001 | Conditioning Tone Maximum Motion Index | behavior | 1551 | 108 |  | 0.487 [-0.282, 0.999] |
| IMPC_FEA_095_001 | Conditioning Shock Average Motion Index | behavior | 1551 | 108 |  | 0.586 [0.231, 0.995] |
| IMPC_FEA_096_001 | Conditioning Shock Minimum Motion Index | behavior | 1551 | 108 |  | 0.21 [-0.852, 1] |
| IMPC_FEA_097_001 | Conditioning Shock Maximum Motion Index | behavior | 1551 | 108 |  | 0.719 [0.218, 1] |
| IMPC_FEA_099_001 | Conditioning Post-shock Freezing Time | behavior | 1551 | 135 | 0.09 [0, 0.278] | 0.88 [0.65, 1] |
| IMPC_FEA_103_001 | Conditioning Post-shock Maximum Motion Index | behavior | 1551 | 108 |  | 0.535 [-0.586, 1] |
| IMPC_GRS_008_001 | Forelimb grip strength measurement mean | physiology | 83155 | 4157 | 0.015 [0.005, 0.026] | 0.499 [0.441, 0.551] |
| IMPC_GRS_009_001 | Forelimb and hindlimb grip strength measurement mean | physiology | 83067 | 4158 | 0.038 [0.029, 0.046] | 0.552 [0.507, 0.598] |
| IMPC_GRS_011_001 | Forelimb and hindlimb grip strength normalised against body weight | physiology | 82998 | 4156 |  | 0.461 [0.416, 0.505] |
| IMPC_HWT_008_001 | Heart weight | morphology | 71786 | 3867 | 0.073 [0.063, 0.082] | 0.655 [0.596, 0.713] |
| IMPC_HEM_001_001 | White blood cell count | physiology | 73628 | 3652 | 0.24 [0.173, 0.294] | 0.344 [0.282, 0.4] |
| IMPC_HEM_002_001 | Red blood cell count | physiology | 75119 | 3683 | 0.024 [0.011, 0.035] | 0.196 [0.124, 0.259] |
| IMPC_HEM_003_001 | Hemoglobin | physiology | 75074 | 3683 | 0.004 [0, 0.01] | 0.228 [0.164, 0.288] |
| IMPC_HEM_004_001 | Hematocrit | physiology | 75103 | 3683 |  | 0.402 [0.343, 0.463] |
| IMPC_HEM_008_001 | Platelet count | physiology | 74953 | 3681 | 0.207 [0.15, 0.253] | 0.319 [0.237, 0.397] |
| IMPC_HEM_029_001 | Neutrophil differential count | physiology | 37519 | 1718 |  | 0.481 [0.41, 0.553] |
| IMPC_HEM_030_001 | Neutrophil cell count | physiology | 34421 | 1495 | 0.206 [0.165, 0.26] | 0.386 [0.291, 0.472] |
| IMPC_HEM_031_001 | Lymphocyte differential count | physiology | 38837 | 1763 |  | 0.473 [0.402, 0.545] |
| IMPC_HEM_032_001 | Lymphocyte cell count | physiology | 34256 | 1483 | 0.183 [0.147, 0.222] | 0.287 [0.205, 0.371] |
| IMPC_HEM_033_001 | Monocyte differential count | physiology | 38805 | 1761 |  | 0.405 [0.312, 0.5] |
| IMPC_HEM_034_001 | Monocyte cell count | physiology | 34262 | 1483 | 0.132 [0.081, 0.18] | 0.045 [-0.06, 0.144] |
| IMPC_HEM_035_001 | Eosinophil differential count | physiology | 37821 | 1760 |  | 0.472 [0.342, 0.587] |
| IMPC_HEM_036_001 | Eosinophil cell count | physiology | 34246 | 1483 | 0.145 [0.07, 0.246] | 0.236 [0.108, 0.366] |
| IMPC_HEM_037_001 | Basophil cell count | physiology | 33765 | 1491 | 0.082 [0, 0.204] | 0.36 [0.217, 0.484] |
| IMPC_HEM_038_001 | Basophil differential count | physiology | 37050 | 1764 |  | 0.512 [0.394, 0.63] |
| IMPC_HEM_039_001 | Large Unstained Cell (LUC) count | physiology | 17501 | 703 | 0.278 [0.167, 0.45] | 0.68 [0.564, 0.792] |
| IMPC_HEM_040_001 | Large Unstained Cell (LUC) differential count | physiology | 17498 | 702 |  | 0.655 [0.505, 0.793] |
| IMPC_IMM_001_001 | Spleen weight | morphology | 6040 | 616 | 0.347 [0.306, 0.387] | 0.398 [0.227, 0.558] |
| IMPC_IMM_002_001 | Percentage of live gated events in Panel A | immunology | 4140 | 565 |  | 0.304 [-0.689, 0.988] |
| IMPC_IMM_003_001 | T cells (panel A) | immunology | 4422 | 549 | 0.013 [0, 0.039] | 0.823 [0.532, 1] |
| IMPC_IMM_004_001 | NKT cells (panel A) | immunology | 4421 | 578 | 0.15 [0.057, 0.248] | 0.648 [0.511, 0.774] |
| IMPC_IMM_006_001 | Others | immunology | 4270 | 578 | 0.042 [0.003, 0.079] | 0.741 [0.457, 0.959] |
| IMPC_IMM_007_001 | CD4 T cells | immunology | 4422 | 549 | 0.016 [0, 0.045] | 0.742 [0.45, 0.986] |
| IMPC_IMM_008_001 | CD8 T cells | immunology | 4417 | 549 | 0.018 [0, 0.048] | 0.859 [0.653, 1] |
| IMPC_IMM_009_001 | DN T cells | immunology | 4399 | 546 | 0.281 [0.199, 0.363] | 0.694 [0.465, 0.916] |
| IMPC_IMM_010_001 | DP T cells | immunology | 3194 | 521 | 0.023 [0, 0.072] | 0.617 [0.321, 0.903] |
| IMPC_IMM_011_001 | CD4 NKT cells | immunology | 4269 | 549 | 0.314 [0.172, 0.466] | 0.664 [0.543, 0.779] |
| IMPC_IMM_012_001 | CD8 NKT cells | immunology | 4251 | 546 | 0.062 [0, 0.15] | 0.631 [0.426, 0.839] |
| IMPC_IMM_013_001 | DN NKT cells | immunology | 4251 | 574 | 0.102 [0.018, 0.191] | 0.777 [0.631, 0.933] |
| IMPC_IMM_014_001 | CD4 CD25+ T cells | immunology | 4414 | 549 | 0.052 [0, 0.1] | -0.142 [-0.543, 0.24] |
| IMPC_IMM_015_001 | CD4 CD25- T cells | immunology | 4414 | 549 | 0.016 [0, 0.049] | 0.764 [0.445, 1] |
| IMPC_IMM_016_001 | CD8 CD25+ T cells | immunology | 3733 | 470 | 0.112 [0.031, 0.208] | 0.905 [0.612, 1] |
| IMPC_IMM_022_001 | CD8 CD25+ NKT cells | immunology | 3715 | 467 | 0.117 [0.008, 0.22] | 0.793 [0.234, 1] |
| IMPC_IMM_023_001 | CD8 CD25- NKT cells | immunology | 3715 | 467 | 0.032 [0, 0.095] | 0.592 [0.39, 0.775] |
| IMPC_IMM_025_001 | DN CD25- NKT cells | immunology | 3715 | 466 | 0.192 [0.094, 0.321] | 0.728 [0.528, 0.915] |
| IMPC_IMM_026_001 | Total number of acquired events in Panel A | immunology | 4189 | 577 | 0.013 [0, 0.038] | 0.38 [0.03, 0.698] |
| IMPC_IMM_027_001 | Total number of acquired events in Panel B | immunology | 4198 | 577 | 0.024 [0, 0.064] | 0.794 [0.683, 0.901] |
| IMPC_IMM_028_001 | CD4 CD44+CD62L- T cells | immunology | 4414 | 549 | 0.082 [0.005, 0.147] | 0.715 [0.491, 0.898] |
| IMPC_IMM_029_001 | CD4 CD44+CD62L+ T cells | immunology | 4174 | 533 | 0.053 [0, 0.111] | 0.753 [0.521, 0.967] |
| IMPC_IMM_030_001 | CD4 CD44-CD62L+ T cells | immunology | 3886 | 470 | 0.034 [0, 0.074] | 0.836 [0.457, 1] |
| IMPC_IMM_031_001 | CD4 CD44-CD62L- T cells | immunology | 2491 | 432 | 0.029 [0, 0.083] | 0.66 [-0.076, 1] |
| IMPC_IMM_032_001 | CD8 CD44+CD62L- T cells | immunology | 4414 | 549 | 0.029 [0, 0.082] | 0.74 [0.55, 0.92] |
| IMPC_IMM_033_001 | CD8 CD44+CD62L+ T cells | immunology | 4414 | 549 | 0.045 [0, 0.104] | 0.751 [0.598, 0.89] |
| IMPC_IMM_034_001 | CD8 CD44-CD62L+ T cells | immunology | 4414 | 549 | 0.019 [0, 0.058] | 0.845 [0.66, 1] |
| IMPC_IMM_035_001 | CD8 CD44-CD62L- T cells | immunology | 3192 | 521 | 0.051 [0, 0.126] | 0.609 [0.395, 0.796] |
| IMPC_IMM_036_001 | DN CD44+CD62L- T cells | immunology | 4243 | 545 | 0.377 [0.261, 0.508] | 0.741 [0.559, 0.911] |
| IMPC_IMM_038_001 | DN CD44-CD62L+ T cells | immunology | 4243 | 545 | 0.315 [0.233, 0.402] | 0.711 [0.421, 0.96] |
| IMPC_IMM_039_001 | DN CD44-CD62L- T cells | immunology | 3173 | 517 | 0.405 [0.312, 0.496] | 0.797 [0.543, 0.995] |
| IMPC_IMM_040_001 | CD4 CD44+CD62L- NKT cells | immunology | 4249 | 547 | 0.365 [0.187, 0.55] | 0.678 [0.557, 0.794] |
| IMPC_IMM_041_001 | CD4 CD44+CD62L+ NKT cells | immunology | 4261 | 549 | 0.04 [0, 0.111] | 0.385 [0.15, 0.592] |
| IMPC_IMM_042_001 | CD4 CD44-CD62L+ NKT cells | immunology | 4261 | 549 | 0.122 [0, 0.317] | -0.115 [-0.507, 0.315] |
| IMPC_IMM_043_001 | CD8 CD44+CD62L- NKT cells | immunology | 4243 | 546 | 0.038 [0, 0.112] | 0.517 [0.205, 0.857] |
| IMPC_IMM_044_001 | CD8 CD44+CD62L+ NKT cells | immunology | 4243 | 546 | 0.096 [0, 0.197] | 0.596 [0.426, 0.773] |
| IMPC_IMM_045_001 | CD8 CD44-CD62L+ NKT cells | immunology | 4243 | 546 | 0.065 [0, 0.192] | 0.257 [-0.16, 0.641] |
| IMPC_IMM_047_001 | DN CD44+CD62L+ NKT cells | immunology | 4243 | 545 | 0.028 [0, 0.083] | 0.763 [0.62, 0.9] |
| IMPC_IMM_048_001 | DN CD44-CD62L+ NKT cells | immunology | 4243 | 545 | 0.644 [0.328, 1.087] | 0.155 [-0.798, 0.911] |
| IMPC_IMM_050_001 | Neutrophils | immunology | 4265 | 449 | 0.047 [0, 0.155] | 0.433 [0.2, 0.647] |
| IMPC_IMM_051_001 | Monocytes | immunology | 4245 | 448 | 0.046 [0, 0.11] | 0.29 [0.068, 0.501] |
| IMPC_IMM_052_001 | Eosinophils | immunology | 4265 | 449 | 0.083 [0.007, 0.153] | 0.366 [0.063, 0.656] |
| IMPC_IMM_053_001 | NK Cells (panel B) | immunology | 3872 | 505 | 0.023 [0, 0.067] | 0.62 [0.461, 0.768] |
| IMPC_IMM_054_001 | NK Subsets (Q1) | immunology | 4169 | 510 | 0.105 [0.019, 0.194] | -0.094 [-0.309, 0.111] |
| IMPC_IMM_055_001 | NK Subsets (Q2) | immunology | 4169 | 510 | 0.041 [0, 0.116] | 0.664 [0.504, 0.829] |
| IMPC_IMM_056_001 | NK Subsets (Q3) | immunology | 3649 | 409 | 0.212 [0.097, 0.364] | 0.481 [0.27, 0.669] |
| IMPC_IMM_057_001 | NK Subsets (Q4) | immunology | 3649 | 409 | 0.056 [0, 0.128] | 0.586 [0.426, 0.734] |
| IMPC_IMM_058_001 | NKT Cells (panel B) | immunology | 3872 | 502 | 0.191 [0.105, 0.285] | 0.843 [0.725, 0.955] |
| IMPC_IMM_059_001 | NKT Subsets (Q1) | immunology | 4169 | 510 | 0.091 [0.027, 0.161] | 0.751 [0.599, 0.889] |
| IMPC_IMM_060_001 | NKT Subsets (Q3) | immunology | 3646 | 407 | 0.353 [0.223, 0.523] | 0.571 [0.407, 0.731] |
| IMPC_IMM_061_001 | T Cells (panel B) | immunology | 4265 | 576 | 0.013 [0, 0.039] | 0.761 [0.628, 0.884] |
| IMPC_IMM_066_001 | Follicular B Cells | immunology | 2561 | 460 | 0.029 [0, 0.068] | 0.886 [0.741, 0.992] |
| IMPC_IMM_068_001 | Transitional B Cells | immunology | 1601 | 317 | 0.137 [0, 0.318] | 0.058 [-0.616, 0.583] |
| IMPC_IMM_070_001 | MZB | immunology | 1165 | 221 | 0.036 [0, 0.117] | 0.836 [0.614, 1] |
| IMPC_IMM_071_001 | MZB (CD21/35 high) | immunology | 2200 | 224 | 0.061 [0, 0.174] | 0.858 [0.594, 1] |
| IMPC_IMM_072_001 | cDCs | immunology | 4260 | 577 | 0.194 [0.12, 0.278] | 0.529 [0.33, 0.71] |
| IMPC_IMM_073_001 | cDCs CD11b Type | immunology | 4260 | 577 | 0.295 [0.195, 0.405] | 0.524 [0.328, 0.702] |
| IMPC_IMM_074_001 | pDCs | immunology | 2569 | 439 | 0.124 [0.051, 0.207] | 0.9 [0.746, 0.991] |
| IMPC_IMM_075_001 | RP Macrophage (F4/80+) | physiology | 951 | 146 | 0.057 [0, 0.114] | 0.637 [0.33, 0.897] |
| IMPC_CAL_008_001 | Total food intake | behavior | 16108 | 876 | 0.027 [0, 0.065] | 0.728 [0.631, 0.822] |
| IMPC_CAL_017_001 | Respiratory Exchange Ratio | physiology | 29638 | 2179 |  | 0.611 [0.501, 0.703] |
| IMPC_CAL_021_001 | Total water intake | behavior | 9823 | 743 | 0.037 [0, 0.09] | 0.491 [0.272, 0.697] |
| IMPC_IPG_010_001 | Fasted blood glucose concentration | physiology | 81056 | 4424 | 0.035 [0.02, 0.05] | 0.399 [0.353, 0.446] |
| IMPC_IPG_011_001 | Initial response to glucose challenge | physiology | 80897 | 4424 | 0.173 [0.142, 0.206] | 0.125 [0.058, 0.195] |
| IMPC_IPG_012_001 | Area under glucose response curve | physiology | 80709 | 4424 | 0.462 [0.406, 0.522] | 0.224 [0.165, 0.278] |
| IMPC_OFD_009_001 | Whole arena average speed | behavior | 70202 | 3367 | 0.072 [0.058, 0.086] | 0.52 [0.475, 0.559] |
| IMPC_OFD_010_001 | Periphery distance travelled | behavior | 69000 | 3332 | 0.09 [0.077, 0.105] | 0.549 [0.503, 0.593] |
| IMPC_OFD_011_001 | Periphery resting time | behavior | 37620 | 1577 | 0.033 [0, 0.055] | 0.66 [0.601, 0.713] |
| IMPC_OFD_012_001 | Periphery permanence time | behavior | 70171 | 3367 | 0.002 [0, 0.005] | 0.644 [0.585, 0.707] |
| IMPC_OFD_014_001 | Center distance travelled | behavior | 68997 | 3332 | 0.054 [0.011, 0.097] | 0.652 [0.606, 0.699] |
| IMPC_OFD_015_001 | Center resting time | behavior | 37617 | 1577 | 0.034 [0, 0.102] | 0.46 [0.353, 0.557] |
| IMPC_OFD_016_001 | Center permanence time | behavior | 70168 | 3367 | 0.013 [0, 0.042] | 0.633 [0.575, 0.697] |
| IMPC_OFD_017_001 | Center average speed | behavior | 64009 | 3088 | 0.074 [0.055, 0.096] | 0.629 [0.553, 0.7] |
| IMPC_OFD_018_001 | Latency to center entry | behavior | 38012 | 1591 | 0.066 [0, 0.203] | 0.441 [0.228, 0.648] |
| IMPC_OFD_019_001 | Number of center entries | behavior | 37999 | 1590 | 0.016 [0, 0.06] | 0.674 [0.613, 0.737] |
| IMPC_OFD_020_001 | Distance travelled - total | behavior | 68018 | 3285 | 0.074 [0.062, 0.088] | 0.566 [0.522, 0.606] |
| IMPC_OFD_021_001 | Number of rears - total | behavior | 49728 | 2537 | 0.04 [0, 0.081] | 0.649 [0.591, 0.701] |
| IMPC_OFD_022_001 | Percentage center time | behavior | 65188 | 3123 |  | 0.633 [0.57, 0.692] |

**Table S2:** List of genotypes with consistently low or consistently high discordant ranks. *Traits* column refers to number of traits that genotype was tested for. *Lower CI* and *upper CI* denote the lower and upper bounds of the Credible Interval associated with the mean discordant rank.

| Background | gene | allele | zygosity | traits | mean discordant rank | lower CI | upper CI |
| --- | --- | --- | --- | --- | --- | --- | --- |
| involves: C57BL/6N | wildtype | wildtype | homozygote | 174 | 0.2634 | 0.2432 | 0.2833 |
| involves: C57BL/6NTac | wildtype | wildtype | homozygote | 186 | 0.2977 | 0.2826 | 0.3141 |
| involves: C57BL/6NJ | wildtype | wildtype | homozygote | 148 | 0.3375 | 0.3105 | 0.3662 |
| involves: C57BL/6NCrl | wildtype | wildtype | homozygote | 192 | 0.3560 | 0.3336 | 0.3776 |
| involves: C57BL/6N;C57BL/6NTac | wildtype | wildtype | homozygote | 170 | 0.4501 | 0.4202 | 0.4784 |
| involves: C57BL/6N | MGI:1338891 | MGI:5793070 | homozygote | 116 | 0.5479 | 0.5019 | 0.5940 |
| involves: C57BL/6N | MGI:3045306 | MGI:6120806 | homozygote | 116 | 0.5520 | 0.5027 | 0.6013 |
| involves: C57BL/6NTac | MGI:3583900 | MGI:5548895 | homozygote | 106 | 0.5552 | 0.5043 | 0.6068 |
| involves: C57BL/6NTac | MGI:1096391 | MGI:5636923 | homozygote | 80 | 0.5575 | 0.5015 | 0.6143 |
| involves: C57BL/6NCrl | MGI:1098802 | MGI:5754588 | heterozygote | 89 | 0.5599 | 0.5020 | 0.6182 |
| involves: C57BL/6NCrl | MGI:1923520 | MGI:5605796 | heterozygote | 127 | 0.5606 | 0.5164 | 0.6058 |
| involves: C57BL/6N | MGI:1891692 | NULL-87B389A49 | homozygote | 58 | 0.5671 | 0.5034 | 0.6338 |
| involves: C57BL/6N | MGI:1920412 | NULL-B804249AA | homozygote | 79 | 0.5671 | 0.5053 | 0.6281 |
| involves: C57BL/6N | MGI:88586 | MGI:5605834 | homozygote | 64 | 0.5712 | 0.5076 | 0.6352 |
| involves: C57BL/6N | MGI:2153041 | MGI:6257715 | homozygote | 52 | 0.5734 | 0.5036 | 0.6491 |
| involves: C57BL/6NCrl | MGI:1923573 | NULL-20A7CB797 | homozygote | 92 | 0.5735 | 0.5122 | 0.6309 |
| involves: C57BL/6N;C57BL/6NTac | MGI:1922656 | MGI:5609354 | heterozygote | 57 | 0.5775 | 0.5082 | 0.6447 |
| involves: C57BL/6N | MGI:1927170 | MGI:5605804 | heterozygote | 57 | 0.5789 | 0.5105 | 0.6461 |
| involves: C57BL/6N | MGI:1935037 | MGI:5568482 | homozygote | 63 | 0.5792 | 0.5148 | 0.6417 |
| involves: C57BL/6N | MGI:2444798 | MGI:5548831 | homozygote | 102 | 0.5799 | 0.5311 | 0.6304 |
| involves: C57BL/6N | MGI:88468 | MGI:5575901 | homozygote | 59 | 0.5812 | 0.5119 | 0.6492 |
| involves: C57BL/6NCrl | MGI:2148202 | MGI:6158484 | homozygote | 73 | 0.5816 | 0.5242 | 0.6408 |
| involves: C57BL/6NCrl | MGI:3613666 | MGI:6158463 | homozygote | 73 | 0.5853 | 0.5310 | 0.6432 |
| involves: C57BL/6N | MGI:2385088 | MGI:5602831 | heterozygote | 59 | 0.5932 | 0.5286 | 0.6570 |
| involves: C57BL/6N | MGI:1922915 | MGI:5603351 | homozygote | 57 | 0.5941 | 0.5215 | 0.6639 |
| involves: C57BL/6N | MGI:1341272 | MGI:5561558 | homozygote | 57 | 0.5994 | 0.5357 | 0.6667 |
| involves: C57BL/6N | MGI:96549 | MGI:5766766 | homozygote | 62 | 0.5995 | 0.5329 | 0.6602 |
| involves: C57BL/6NTac | MGI:1202301 | MGI:5692595 | homozygote | 78 | 0.6014 | 0.5456 | 0.6621 |
| involves: C57BL/6NTac | MGI:104993 | MGI:5692557 | homozygote | 54 | 0.6094 | 0.5386 | 0.6742 |
